# Geographical Variability of Mineral Elements and Stability of Restrictive Mineral Elements in Terrestrial Cyanobacteria Across Gradients of Climate, Soil, and Atmospheric Wet Deposition Mineral Concentration

**DOI:** 10.3389/fmicb.2020.582655

**Published:** 2021-01-18

**Authors:** Weibo Wang, Hua Li, René Guénon, Yuyi Yang, Xiao Shu, Xiaoli Cheng, Quanfa Zhang

**Affiliations:** ^1^CAS Key Laboratory of Aquatic Botany and Watershed Ecology, Wuhan Botanical Garden, Chinese Academy of Sciences, Wuhan, China; ^2^Center of Plant Ecology, Core Botanical Gardens, Chinese Academy of Sciences, Wuhan, China; ^3^CAS Key Laboratory of Algal Biology, Institute of Hydrobiology, Chinese Academy of Sciences, Wuhan, China; ^4^EPHOR, Institut Agro, Angers, France

**Keywords:** terrestrial cyanobacteria, geographic variation, mineral elements, climate, soil nutrients, atmospheric wet deposition, restrictive element stability hypothesis, environmental adaptation

## Abstract

Terrestrial cyanobacteria *Nostoc commune* is an ideal species to study the geographical variation of mineral elements of soil cyanobacteria at the species level. Here, we first address the following questions: (1) from where are these mineral elements, (2) are there geographical variations for these mineral elements, and if so, (3) which environmental factors drive the geographical variation of these mineral elements? Second, we tested whether the soil cyanobacterial mineral elements followed the “restrictive element stability hypothesis” of higher plants. Finally, we explored the effect of mineral geographic variation on ecological adaptation of soil cyanobacteria. We collected *N. commune* samples across gradients of climate, soil, and atmospheric wet deposition mineral concentration in mainland China. We measured fifteen minerals, including five macroelements (N, Ca, K, Fe, P), five microelements (Mn, Zn, Cu, Co, Se), and five heavy metals (Pb, Cr, As, Cd, Hg). We found that five elements (P, Cu, Zn, Co, Pb) had significant geographical variation. They increased as the distance from the equator increased and decreased as the distance from the prime meridian increased. Mean annual precipitation and mean annual temperature explained most of the variation. We did not find any significant correlations between the mineral element contents in *N. commune* and the minerals in soil and rainfall, except for P. There was no significant correlation between the variation coefficients of different elements and their actual detected contents and their potential physiological required contents. The statistical results of our experiment did not support the “restrictive element stability hypothesis.” We speculated that net accumulation of mineral elements in cyanobacterial cells and extracellular polysaccharides (EPS) might play an important role for terrestrial cyanobacteria in the adaptation to dry and cold conditions.

## Introduction

Recently, significant progress has been achieved in terrestrial microbial biogeography (Fierer and Jackson, 2006; Ranjard et al., 2010, 2013). However, compared to aquatic microbial biogeography (Quigg et al., 2003; Garcia et al., 2016; Godwin and Cotner, 2018) and the biogeography of large plants and animals (both aquatic and terrestrial) (Humphries and Parenti, 1999; Dormann et al., 2007), studies of terrestrial microbial biogeography remain relatively rare. For aquatic microorganisms and higher plants and animals, the geographical variation of their community structure and the variation of their biochemical composition and mineral elements (stoichiometric characteristics) can be studied, both at the community level (Han et al., 2005, 2011; Yvon-Durocher et al., 2015) and the species level (Zhou et al., 2013; Garcia et al., 2016; Brady and Seth, 2017; Godwin and Cotner, 2018). For example, biogeography and variability of mineral elements in higher plant leaves have been well-studied and the “restrictive element stability hypothesis” has been posed (Han et al., 2005, 2011). Studies on terrestrial microbial geography mainly focus on the geographical variation of microbial community structure (Garcia-Pichel et al., 2013), while geographical variations in its biochemical composition and mineral elements (stoichiometric characteristics) are rarely reported.

The main limiting factor for the study of geographical variations in terrestrial microbial biochemical composition and mineral elements is the availability of adequate microbial biomass. Microbial biomass in soil at both the community and species levels has been difficult to determine. The separation of soil microorganisms from soil particles and organic matter is difficult. Even if a small amount of biomass can be obtained, its content is too small to carry out detailed biochemical composition analysis or mineral element analysis. On the other hand, the biochemical composition and mineral elements of microbial biomass from artificial propagation may change relative to naturally growing microorganisms due to the difference in its environment (Dawson, 1919).

*Nostoc commune*, a form species, is one of the few soil microorganisms that can gain a large amount of biomass in its natural state. Along with two other form species, *Nostoc flagelliforme* and *Nostoc sphaeroids*, it is usually considered as belonging to the same genetic species (Wright et al., 2001). *N. commune* live mainly on the soil surface and are geographically distributed from polar valleys to tropical regions (Dodds et al., 1995). It is a filamentous cyanobacteria, which not only falls within prokaryotes, but also has chlorophyll and can carry out photosynthesis (Dodds et al., 1995). In the natural state, the filaments of *N. commune* and their secreted extracellular polysaccharides (EPS) can form a colony structure with large biomass, and is a traditional food in China, East Asia, and some African countries (Briones-Nagata et al., 2007) (Supplementary Figure 1).

China has large gradients of vegetation and has north-south and east-west gradients in climate, soil substrate materials, and atmospheric wet deposition mineral concentrations (Han et al., 2011; Zhu et al., 2016a,b). *N. commune* can be found in almost all areas of China and achieves a large biomass, so it is a great candidate for the study of soil microbial geography and of biochemical composition and mineral elements (stoichiometric characteristics) at the species level.

**Figure 1:**
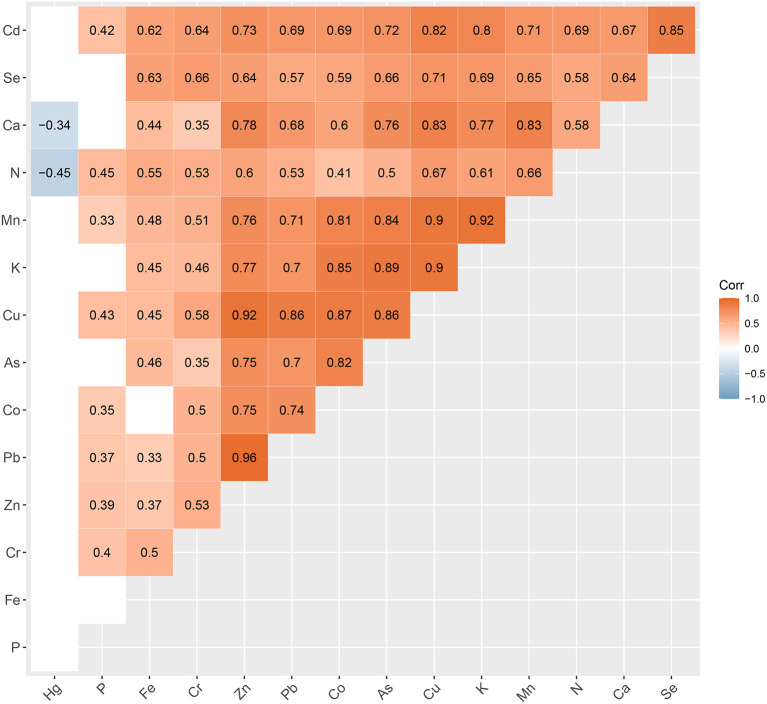
Correlation heat map of the 15 elements analyzed in our study. Levels of statistical significance *p* < 0.05 are not shown.

In the present study, we focused on 15 mineral elements of *N. commune* (Figure 1) and investigated the geographical variation of mineral elements of soil microorganisms at the species level in mainland China. First, we addressed the following questions: (1) from where are these mineral elements, (2) are there geographical variations for these mineral elements in *N. commune* like those of higher plants or aquatic algae, and (3) which environmental factors dominate the geographical variation of these mineral elements? Second, we tested whether the soil cyanobacterial mineral elements followed the higher plants “restrictive element stability hypothesis” (Han et al., 2011), which implies that variability is lowest for elements that are required in the highest concentrations. We divided the 15 mineral elements according to their physiological necessity: macroelements, microelements, and heavy metals (Supplementary Figure 1). Finally, we explored the effect of geographic variation of mineral element content on the ecological adaptation of soil cyanobacteria.

## Materials and Methods

### Sample Collection and Pretreatment

We collected *N. commune* samples from 30 different counties, distributed in 16 provinces or autonomous regions in mainland China (Supplementary Figure 2, Supplementary Table 1). To minimize the influence of seasonal aspect on the variation of sample characteristics, all the samples were collected in the late spring in 2011 and 2012. In each sampling area, 5–10 samples from different microclimates and soil textures were collected to make one composite sample to minimize the heterogeneity of the microenvironment. For each composite sample, more than one kilogram of dry matter was collected to minimize the influence of life-history differences of the samples. We obtained a total of 33 composite samples for our study. All fresh samples were washed with distilled water, air dried, and stored in the desiccators for further analyses.

### Biochemical Composition and Mineral Content Analysis

We measured crude protein and ash according to the methods prescribed by the Chinese Standard Agency for food as described by Hao et al. (2011) (Supplementary Table 2). More specifically, crude protein was estimated using the Kjeldahl method and ash content was expressed as the percentage of residue remaining after dry oxidation at 550 ± 25°C (Hori et al., 1990). Total organic carbon (TOC) was measured by the potassium dichromate method (Supplementary Table 2).

The total nitrogen (N) in the *N. commune* was measured using the semi-micro Kelvin method (Supplementary Table 3). The total phosphorus (P) was determined by molybdenum blue colorimetry (Supplementary Table 3). Potassium (K), copper (Cu), zinc (Zn), iron (Fe), manganese (Mn), cobalt (Co), selenium (Se), calcium (Ca), lead (Pb), cadmium (Cd), and chromium (Cr) were measured following the procedure previously described by Tamasi et al. (2013) with flame atomic absorption spectrophotometers Perkin-Elmer 5000 and Perkin-Elmer 300 (Perkin-Elmer, Monza, Italy) (Supplementary Table 3). Mercury (Hg) concentrations were analyzed following the U.S. EPA Method 1630, including pre-digestion with 0.5% (v/v) 0.2 N bromine monochloride, reduction with hydroxylamine hydrochloride, further reduction with tin chloride, purging of Hg onto gold traps, and quantification of Hg by cold vapor atomic fluorescence spectrometry (CVAFS, Tekran Model 2,500 Hg Analyzer, Knoxville, USA) (Rothenberg et al., 2012). For quantification of arsenic content, dry matter samples were predigested with a mixture of nitric acid and perchloric acid. Arsenic content in the resulting solution was determined by atomic absorption spectrophotometry (Spectr AA220, Varian Medical Systems, Inc., USA) (de Freitas-Silva et al., 2016).

### Climate Variables, Soil Data, and Atmospheric Deposition Data

In this study, we collected eleven climatic variables (Supplementary Table 4) to analyze the climatic controls on the spatial patterns of the 15 mineral elements and biochemical composition of *N. commune*: mean annual temperature (MAT; °C), mean annual precipitation (MAP; mm), mean annual solar radiation (MAR; MJ m^−2^ day^−1^), mean maximum annual temperature (MATmax; °C), mean minimum annual temperature (MATmin; °C), mean annual precipitation days (MAPD, d), relative humidity (RH, %), frost free days (FFD, d), mean annual evaporation (MAE, mm), drought index (DI), and UV radiation (MJ m^−2^ year^−1^). Climate data for the period 1981–2010 were compiled from the Chinese National Meteorological Information Center.

Mean soil mineral elements concentrations in China were obtained from the national soil survey (http://vdb3.soil.csdb.cn/) (Supplementary Table 5). Mean plant leaf mineral elements concentrations in China were from Han et al. (2011) (Supplementary Table 5). Soil TOC, N, P, C/N, N/P, and K data for each sample area were obtained from the national soil survey (http://vdb3.soil.csdb.cn/), and other soil minerals were from the soil pollution condition investigation communiqué (Environmental Protection Department of and The Ministry of Land and Resources of the People' s Republic of China, 2014; Chen et al., 2015) (Supplementary Table 6).

Mean atmospheric total deposition data for mineral concentrations were obtained from Pan and Wang (2014) (Supplementary Table 7). The mean atmospheric wet deposition data for mineral concentrations were obtained from observation by Michaelis (1997). Part of the chemical characteristics of wet deposition (Ca, K) for our sample areas were obtained from Xie and Xue (2012) (Supplementary Table 8). The other wet deposition minerals (N, P, Cr, Pb, Cd), pH, and total salinity were estimated using the Kriging extrapolation method by the software ArcGIS 10, and the original data were from the Chinese Ecosystem Research Network (CERA) (Zhu et al., 2016a,b) (Supplementary Table 8).

### Data Analysis

All *N. commune* mineral concentrations were log_10_-transformed before analyses to improve the normality of the data. As the protein concentration is relatively stable, elemental concentrations relative to protein were used as an index of stoichiometry. The preliminary statistical analyses were first performed to screen the environmental factors. Those factors (MAPD, RH, FFD, MAE, and DI) that had high collinearity (VIF > 10) with other environmental factors and those (altitude; UV radiation; soil TOC, C/N, and N/P) that had no significant correlations with the mineral elements of *Nostoc commune* were removed from the next statistical analyses. To explore the possible geographic variation of Nostoc minerals, we performed Pearson correlation and Spearman's rank correlation between Nostoc minerals and geographic characteristics (distance from the equator and distance from the prime meridian).

Linear regressions and stepwise multiple regressions were used to determine the most influential climate variables among the five climate variables (MAP, MAT, MAR, MAT_max_, MAT_min_). Pearson correlation and Spearman's rank correlation were performed between Nostoc minerals and MAP and MAT. To explore the possible effects of soil and atmospheric wet deposition, Spearman's rank correlation was performed between Nostoc minerals and the corresponding soil mineral contents and the mineral concentrations of atmospheric wet deposition.

We conducted redundancy analysis (RDA) to study the relationship between elements of significant geographic variation and their related environment variables. Variation partitioning was performed to explain the importance of climate variables, soil variables and atmospheric wet deposition variables. Meanwhile, for each mineral element which had significant geographic variation, stepwise multiple regressions were implemented to identify the most influential environmental variables.

All analyses were performed with statistical software IBM SPSS 23 (IBM Corp, Armonk) and R 4.0.2 (R Development Core Team, 2020) using the “vegan” and “ggplot2” packages and custom scripts.

## Results

### Statistics and Mineral Source Analysis

The 15 mineral elements in this study include 5 macroelements (N, Ca, K, Fe, P), 5 microelements (Mn, Zn, Cu, Co, Se), and 5 heavy metals (Pb, Cr, As, Cd, Hg) (Supplementary Figure 3). The mean values of 15 mineral elements varied greatly, from 0.02 mg kg^−1^ dry weight of Hg to 261.79 g kg^−1^ dry weight of N (Supplementary Table 9).

The content of some heavy metals was much higher than that of trace elements, such as the contents of Pb (15.66 mg kg^−1^) and Cr (14.74 mg kg^−1^) were higher than Cu (9.12 mg kg^−1^), as (4.01 mg kg^−1^) was higher than Co (2.94 mg kg^−1^), and Cd (0.40 mg kg^−1^) was higher than Se (0.20 mg kg^−1^) (Supplementary Figure 3, Supplementary Table 9). There were significant positive correlations between the contents of some mineral elements, especially the divalent cations Zn, Cu, and Pb, and their correlation coefficients were all over 0.80 (Pb & Zn, *r* = 0.93; Pb & Cu, *r* = 0.88; Zn & Cu, *r* = 0.90) (Figure 1).

**Figure 2:**
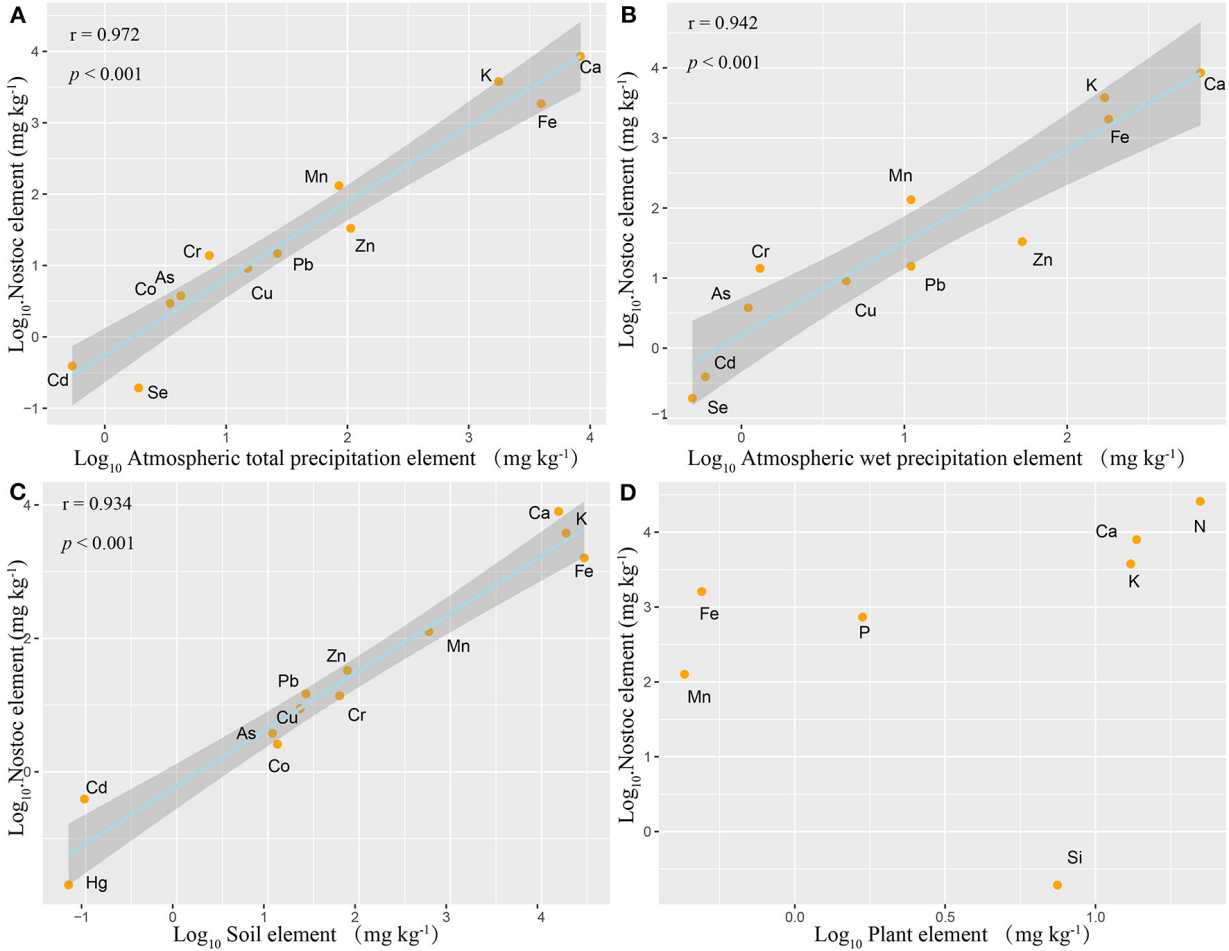
Relationships between mean *N. commune* and plant leaf mineral concentration and atmospheric deposition and soil mineral concentration. **(A)** Nostoc vs. Atmospheric total deposition, Log_10_(y) = 1.069 Log_10_(x)−0.258 (*r*^2^ = 0.972, *p* < 0.001); **(B)** Nostoc vs. Atmospheric wet deposition, Log_10_(y) = 0.669 Log_10_(x)−0.007 (*r*^2^ = 0.942, *p* < 0.001); **(C)** Nostoc vs. Soil, Log_10_(y) = 0.861 Log_10_(x)−0.213 (*r*^2^ = 0.934, *p* < 0.001); **(D)** Nostoc vs. Plant leaf. Ninety fine percentage of confidence bands for all fitted curves are also shown.

The element ratio of *N. commune* was similar to the compositions of atmospheric total deposition minerals (*r*^2^ = 0.97, *p* < 0.001) and wet deposition minerals (*r*^2^ = 0.94, *p* < 0.001) (Figures 2A,B), and is similar to the composition of soil minerals (*r*^2^ = 0.96, *p* < 0.001) (Figure 2C). However, the element ratio of *N. commune* is completely different than that of higher plants (Figure 2D), and the element ratio of higher plants and the soil in which they grow is also different.

### Biogeographic Patterns of Nostoc Minerals and Environmental Influence

**Figure 3:**
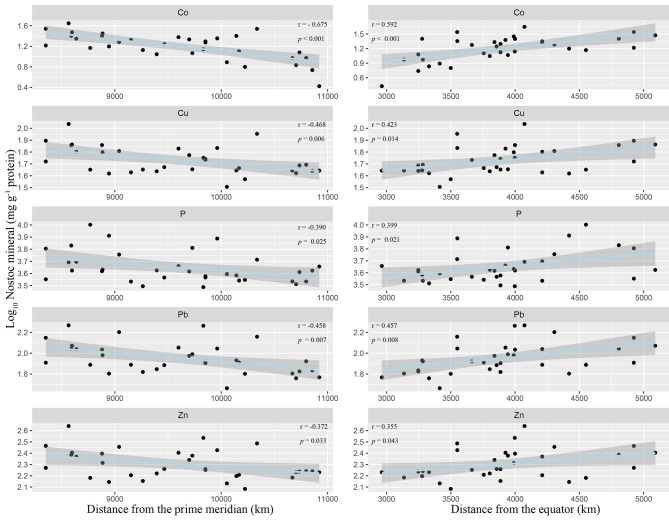
Geographic patterns of *N. commune* minerals in China. Five minerals show significant latitudinal (distance from the equator at certain longitude) and longitudinal (distance from the prime meridian at certain latitude) linear correlations (*p* < 0.05).

Among the 15 elements, 5 elements (P, Cu, Zn, Co, Pb) showed significant geographical variation (Figure 3, Supplementary Table 10). They increased with the increase of the distance from the equator and decreased with the increase of the distance from the prime meridian (Supplementary Table 10). What is noteworthy is that the increase of these elements was not accompanied by the decrease of other elements. Meanwhile, the ash content showed similar geographical variation (Supplementary Table 10), and the five elements (K, Cu, Zn, Co, Pb) were positively correlated with the ash content (Supplementary Figure 4).

Six minerals (P, K, Cu, Zn, Co, Pb) were significantly and negatively correlated with MAP (Table 1, Supplementary Table 11, Supplementary Figure 5), and 5 (P, Cu, Zn, Co, Pb) of these were also significantly and negatively correlated with MAT (Table 1, Supplementary Table 11). In addition to MAP and MAT, we also introduced MAR, MAT_max_, and MAT_min_ climate variables to conduct a stepwise regression analysis for these 6 elements (Supplementary Table 12). The results showed that for most elements (P, K, Zn, Co, Pb), MAP could explain all the variations, and other climate factors were removed in the stepwise regression process. But for Cu, MAT can explain all the variations, and other climate factors were removed in the stepwise regression process.

**Table 1:** Correlations between Nostoc biochemicals and minerals with climate variables.

**Biochemicals and minerals (mg g^**−1**^protein)**	**MAP (mm)**				**MAT (**^****°****^**C)**			
	**Pearson correlation**		**Spearman's rank correlation**		**Pearson correlation**		**Spearman's rank correlation**	
	***r***	***p***	**ρ**	***p***	***r***	***p***	**ρ**	***p***
Ash	−0.332	0.068	−0.380	0.035^*^	−0.380	0.035^*^	−0.435	0.014^*^
TOC	−0.063	0.728	−0.241	0.176	−0.025	0.892	−0.219	0.221
N	−0.001	0.997	0.003	0.985	0.076	0.673	0.015	0.932
Ca	0.036	0.841	−0.019	0.918	−0.103	0.569	−0.099	0.584
K	−0.353	0.044^*^	−0.361	0.039^*^	−0.311	0.078	−0.354	0.043^*^
Fe	0.180	0.316	0.154	0.392	0.227	0.205	0.232	0.194
P	−0.405	0.019^*^	−0.429	0.013^*^	−0.380	0.029^*^	−0.393	0024^*^
Mn	−0.277	0.118	−0.341	0.052	−0.319	0.070	−0.371	0.034^*^
Zn	−0.383	0.028^*^	−0.347	0.048^*^	−0.364	0.037^*^	−0.393	0.024^*^
Cu	−0.449	0.009^**^^‘^	−0.424	0.014^*^	−0.482	0.005^**^	−0.471	0.006^**^
Co	−0.636	0.000^***^	−0.584	0.000^***^	−0.605	0.000^***^	−0.617	0.000^***^
Se	−0.017	0.923	−0.118	0.513	−0.200	0.265	−0.239	0.181
Pb	−0.474	0.005^**^	−0.423	0.014^*^	−0.418	0.015^*^	−0.431	0.012^*^
Cr	−0.327	0.063	−0.274	0.123	−0.252	0.157	−0.171	0.341
As	−0.296	0.094	−0.286	0.107	−0.326	0.064	−0.262	0.140
Cd	−0.200	0.265	−0.149	0.408	−0.221	0.217	−0.142	0.430
Hg	0.017	0.923	−0.175	0.329	−0.023	0.898	−0.154	0.392

*Levels of statistical significance*:

* *p < 0.05*,

** *p < 0.01*,

and

*** *p < 0.001*.

Spearman rank's correlation were conducted for the content of different mineral elements in *N. commune* and their corresponding soil elements, and the results showed that only the content of P was significantly and positively correlated with the content of soil P, while the other elements were not significantly correlated or were negatively correlated (Table 2). Among the 15 elements analyzed, the contents of Co and Pb were significantly and positively correlated with soil pH (Supplementary Table 13).

**Table 2:** Spearman's rank correlations (ρ) between Nostoc minerals and soil minerals and atmospheric wet precipitation minerals.

***N* = 33**	**Soil minerals**		**Atmospheric wet precipitation minerals**	
	**ρ**	***p***	**ρ**	***p***
N	−0.074	0.683	0.182	0.311
Ca	NA	NA	−0.057	0.753
K	0.281	0.147	0.131	0.468
P	0.351	0.045^*^	−0.339	0.054
Zn	−0.409	0.018^*^	NA	NA
Cu	−0.534	0.001^**^	NA	NA
Pb	−0.378	0.030^*^	−0.521	0.002^**^
Cr	−0.020	0.914	−0.164	0.361
As	0.084	0.641	NA	NA
Cd	−0.029	0.871	−0.132	0.463
Hg	−0.160	0.373	NA	NA

*Nostoc minerals (mg g^−1^ protein), soil minerals (mg g^−1^), and atmospheric wet precipitation minerals [N, Ca, K, P (μmol l^−1^), Pb, Cr, Cd (mg m^−2^)] are log_10_-tranformed before analysis. Levels of statistical significance*:

* *p < 0.05*,

** *p < 0.01*.

We also conducted Spearman rank's correlation for the content of different mineral elements in *N*. *commune* and their corresponding atmospheric wet deposition elements. We found that no element was significantly and positively correlated with the content of the corresponding wet deposition elements (Table 2). In addition, no significantly and positively correlations were found between the content of mineral elements and the total salinity of atmospheric wet deposition. However, among the 15 elements analyzed, 3 elements (Zn, Cu, Pb) were positively correlated with rainfall pH (Supplementary Table 13).

**Figure 4:**
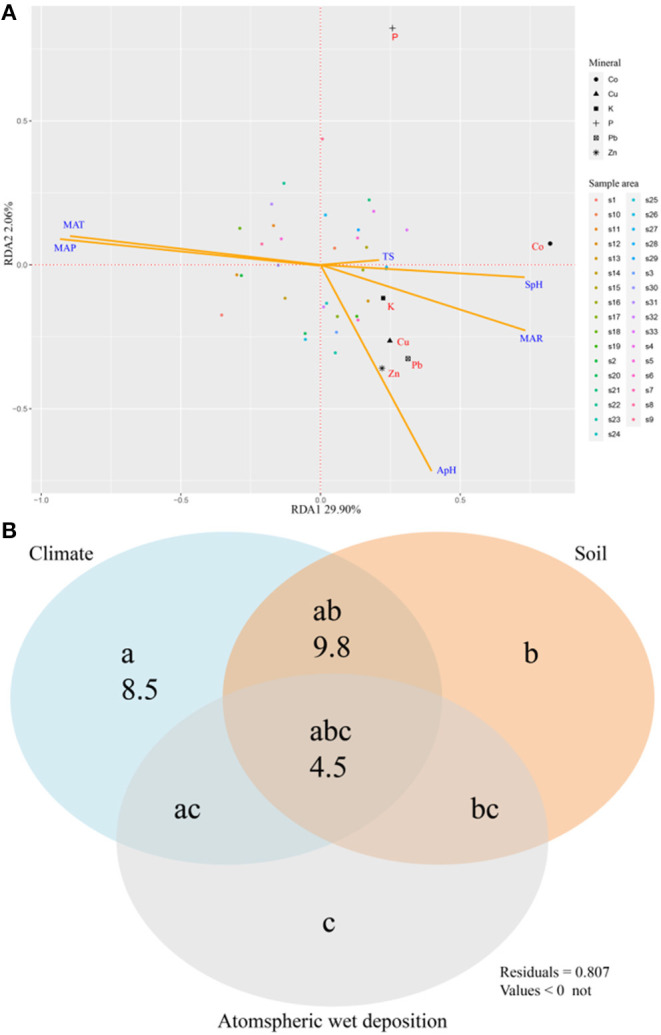
Redundancy analysis showing 6 mineral concentrations of *N. commune* in relation to environmental factors **(A)** and variation partitioning (*r*^2^) of environmental factors in according for the variation in 6 mineral concentrations **(B)**. The symbols a, b, c represent the independent effects of climate (MAP, MAT, MAR), soil (soil pH, SpH), and atmospheric wet deposition (rainfall pH, ApH; rainfall total salinity, TS), respectively; ab is the interactive effect of climate and soil; ac is the interactive effect of climate and atmospheric wet deposition; bc is the interactive effect of soil and atmospheric wet deposition, and abc is the interactive effect of climate, soil, and atmospheric wet deposition.

RDA analysis was used to study the relationships between 6 elements (P, K, Cu, Zn, Co, Pb) and their associated environmental factors, and we found that MAP and MAP were the most important environmental factors (Figure 4A). The variance partitioning showed that environmental factors can explain 22.8% of the variation, the variation is mainly explained by climate factors (8.5%), the interactive effect of climate and soil (9.8%), and the interactive effect of climate, soil and atmospheric wet precipitation (4.5%) (Figure 4B). Stepwise regression analysis was then conducted (Table 3), and the results showed that MAT explained all the variations of the four elements P, Zn, Co, and Pb, while MAT explained all the variations for Cu.

**Table 3:** Linear regressions of *N. commune* minerals vs. climate, soil, and atmospheric wet deposition (AWP) characteristics.

	**Climate**		**AWP**		**Soil**	
	**MAP**	**MAT**	**TS**	**pH**	**pH**	**Stepwise multiple Regression (SMR)**
***N*** **=** **33**	**1 Model (Adj. r**^**2**^**)**	**2 Model (Adj. r**^**2**^**)**	**3 Model (Adj. r**^**2**^**)**	**4 Model (Adj. r**^**2**^**)**	**5 Model (Adj. r**^**2**^**)**	**6 Model (Adj. r**^**2**^**) (predictive variable)**
P	0.137	0.117	0.110			0.137 (MAP)
K	0.097					
Zn	0.119	0.104				0.119 (MAP)
Cu	0.176	0.207				0.207 (MAT)
Co	0.384	0.346		0.263		0.384 (MAP)
Pb	0.200	0.148		0.105		0.200 (MAP)

*MAP (mean annual precipitation, mm) enters model 6 (stepwise multiple regression, SMR) before other characteristics, indicating that MAP is more powerful in explaining variations in the Nostoc mineral than is rainfall TS (total salinity), rainfall pH, soil pH. All p < 0.05 in models are shown in the table*.

### Stability of Limiting Mineral Elements

**Figure 5:**
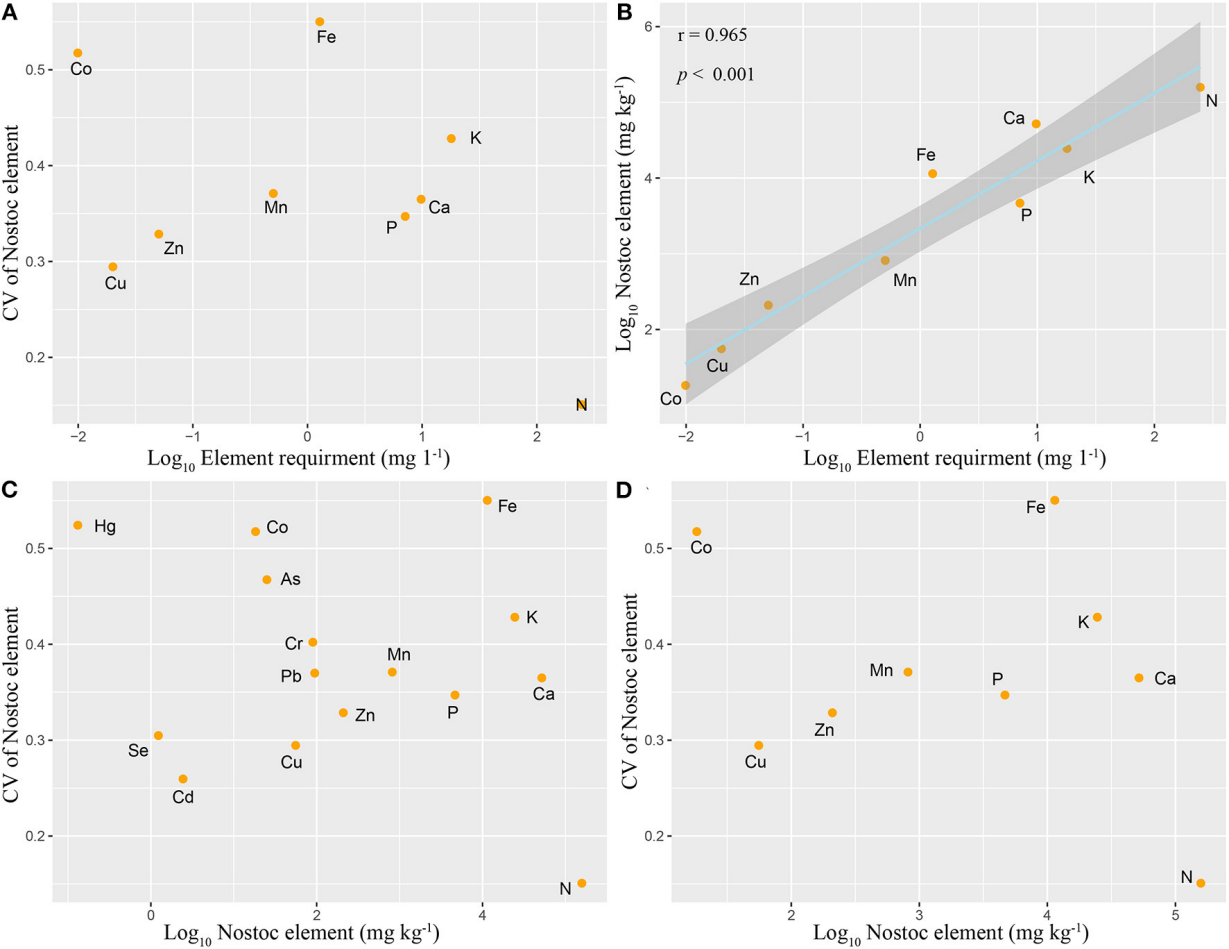
Relationships between *N. commune* elemental requirement, mean *N. commune* elemental concentration, and its coefficient of variation (CV). **(A)** CV vs. Elemental requirement; **(B)** Nostoc element (heavy metals not included) vs. Elemental requirement, Log_10_(y) = 0.893 Log_10_(x) +3.333 (*r*^2^ = 0.965, *p* < 0.001); **(C)** CV vs. Nostoc element (heavy metals included); **(D)** CV vs. Nostoc element (heavy metals not included). Ninety fine percentage of confidence bands for all fitted curves are also shown.

In this study, the element ratio of cyanobacteria BG11 medium (Supplementary Table 14) was selected as the nutrient requirement ratio, macroelements were generally considered as limiting elements, trace elements were considered to be non-limiting elements, and heavy metals were considered to be non-essential elements. The coefficient of variation was used to represent the relative variation (stability) of elements. We found that the element ratio of the samples and nutrient requirement of *N*. *commune* was roughly the same (*r*^2^ = 0.93, *p* < 0.001, Figure 5B). However, the variation coefficients of different elements were not significantly correlated with their measured contents [Figure 5C (heavy metals included); Figure 5D (heavy metals not included)], and they were not significantly correlated with required nutrient contents (Figure 5A).

## Discussion

### Absorption (Intracellular) and Adsorption (Extracellular) of Mineral Elements in Cyanobacteria

**Figure 6:**
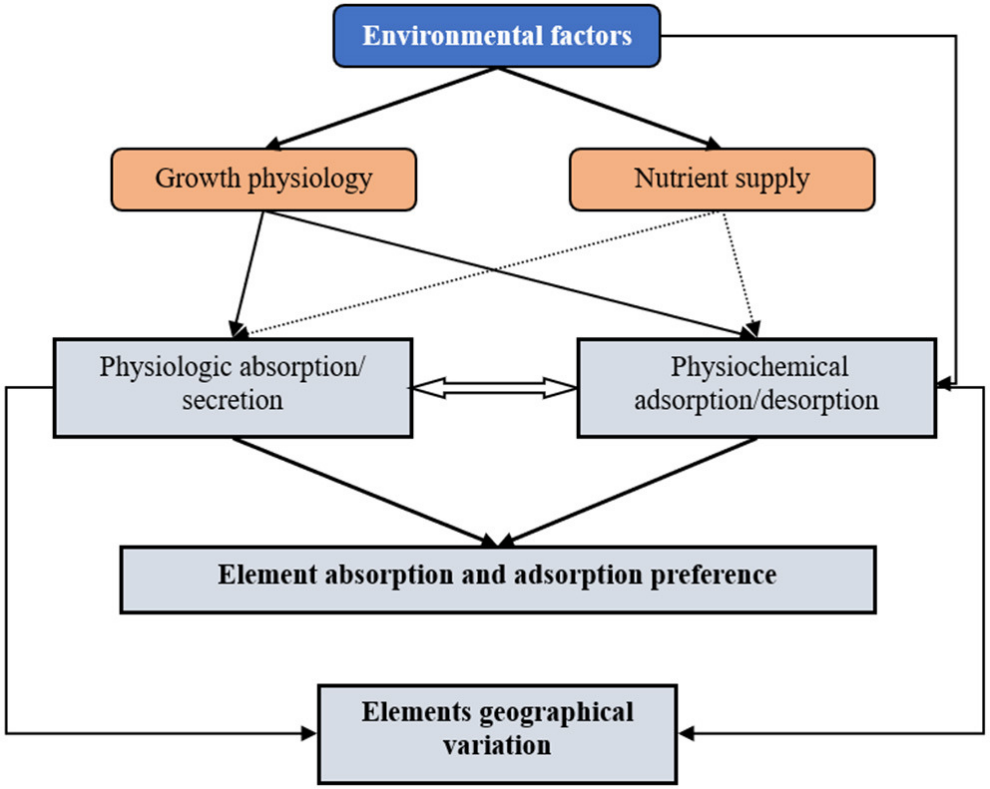
The schematic diagram showing environmental factors control the mineral patterns of *N. commune*. Environmental factors (especially precipitation and temperature) shape the element absorption and adsorption preference and the elements geographical variation of *N. commune*. Environmental factors (especially precipitation and temperature) can indirectly influence physiologic absorption/secretion and physiochemical adsorption/desorption by regulating growth physiology of *N. commune*, and can also directly influence the physiochemical adsorption/desorption of *N. commune*.

The mineral element content in cyanobacteria comes from two sources, the physiological absorption/secretion process and the physiochemical adsorption and desorption process (Volesky and Holan, 1995) (Figure 6). The physiological absorption and secretion process are an active, energy-consuming metabolic process that mainly occurs in the cell membrane and requires that the cyanobacteria have metabolic activity (Gadd, 1990). Some of the physiologically absorbed mineral elements participate in metabolic activities as physiologically essential elements, and some are stored in the cells for reserve (luxury consumption) (Brown and Shilton, 2014). When environmental conditions change, cyanobacterial cells can also secrete mineral elements to maintain osmotic balance or expel toxic and harmful elements (Kaplan, 2013).

Cyanobacteria physiochemical adsorption and desorption is a passive process that mainly occurs in extracellular polymers (EPS), runs by ion exchange or by binding positive metals with negative charged carboxylic groups in EPS (Davis et al., 2003), and does not require the metabolic activity of cyanobacterial cells (Davis et al., 2003). It can also be carried out in dead algal colonies. Mineral physiochemical desorption occurs when the pH value of the water decreases and ion exchange drive this process (Blanco et al., 1998). Mineral elements passively adsorbed by algae can be further actively absorbed by cyanobacterial cells, and the mineral elements actively secreted by algal cells can be passive re-adsorbed in EPS (Fiore and Trevors, 1994; Kaplan, 2013).

### Source Analysis of Mineral Elements in *N. commune*

*N. commune* has no root, stem, or leaf differentiation and no specialized absorption tissue, so it is not possible for them to directly absorb various mineral elements from the soil subsurface (Dodds et al., 1995). The element source may come from rainfall (atmospheric wet deposition), or soil surface runoff induced by rainfall, in which case the element composition is affected by rainfall as well as soil composition. We found that the element ratio of Nostoc elements was remarkably similar to that of atmospheric deposition (total deposition and wet deposition) and soil elements, which confirmed that the mineral elements mainly came from rainfall or the soil. However, the elements ratio did not change during physiological activities, as some minerals were enriched and some were diluted. This conservation of the elemental ratio was different from higher plants, whose elemental ratio is significantly altered according to the soil in which they grow. There may be two reasons for this difference. First, the content of physiochemical and passively adsorbed mineral elements far exceeds physiological demand. The ratio of mineral elements of *N. commune* measured in this study is mainly from the ratio of passively adsorbed mineral elements. Secondly, both the ratio of bioaccumulated mineral elements (physiologically required and stored) and passively adsorbed mineral elements are similar to atmospheric rainfall and soil surface runoff, which is the result of long-term evolution of cyanobacteria. Our results showed that the ratio of essential elements in cyanobacteria (BG11 medium) was the same as the elemental ratio of rainfall (*r*^2^ = 0.78, *p* = 0.002). Therefore, our study was more inclined to support the second reason.

### Geographical Variation of Elements and Environmental Determinants

Among the 15 elements investigated, 5 mineral elements showed significant geographical variation, while the others showed no significant geographical variation. Our results also showed that *N. commune* had a preference for the absorption and adsorption of different mineral elements. These preferences may be related to physiological needs (regional environmental adaptation) of *N. commune*, or the absorption and adsorption process and mechanisms for different mineral elements. For example, P was usually absorbed as a luxury (Brown and Shilton, 2014), while some divalent cation such as Cu, Pb, and Zn are the main elements of passive adsorption (Davis et al., 2003), and Co plays an important role in biological nitrogen fixation of cyanobacteria (De Philippis et al., 2001).

Our results showed that there was no significant positive correlation between the contents of these elements in *N. commune* (except P, who showed a positive correlation with soil P) and their contents in the natural environment (including rainfall and soil), which indicated that passive adsorption was not the main factor determining its geographical variation. However, the contents of 2 and 3 of these 5 elements in *N. commune* were positively correlated with the pH values of rainfall and soil, respectively, indicating that passive desorption may have a certain impact on the geographical variation of mineral elements, because acidic conditions can result in competition between free metal ions and H^+^ for the same uptake sites, leading to an increase in cell desorption (Fiore and Trevors, 1994).

Among all the environmental factors, climatic factors can explain the variation of the geographically variable mineral elements (P, Cu, Zn, Co, Pb), which decreased with decreases in temperature and rainfall. This geographical variation may be related to the adaptation of *N. commune* to drought and low temperature conditions, which is regulated by active physiological demand. In addition to the above five mineral elements with significant geographical variation, the content of K in *N. commune* was also significantly correlated with MAP and MAT. It increased with increased drought and decreased temperature. As K is often a limiting element in the environment, its increase (luxury absorption) under drought or low temperature stress may also be actively regulated by physiological demand (Qiu et al., 2004).

Our results supported the “growth rate hypothesis” (Allen and Gillooly, 2009; Garcia et al., 2016), which implied that all the mineral geographic variation is adjusted by the physiological demand. Due to the mismatching of mineral concentrations of Nostoc with their environments, the results did not support the “environmental nutrient supply hypothesis” (Reich and Oleksyn, 2004). Meanwhile, for *N. commune*, passive desorption determined by environment pH supplied another path for the regulation of its stoichiometry (Figure 6).

### Element Physiological Requirement and Stability of Limiting Elements

We found that the elemental ratio of *N. commune* was basically the same as that of the BG11 medium, indicating that under natural condictions, the physiological requirements of *N. commune* were basically the same as those of other cyanobacteria. There was no particularity for *N. commune*. Therefore, the results of this study can be expanded to other cyanobacteria or even other soil microorganisms.

Depending on the “restrictive element stability hypothesis,” macroelements are usually limiting elements, while microelements are non-limiting elements. Variation of elements is inversely proportional to the element content, that is to say, the variation of largely required elements is small (Han et al., 2011). Our study not only investigated the variability of macroelements and microelements, but also investigated the variability of heavy metals. According to the hypothesis inference, the variability of heavy metals should be the highest because there is no physiological need for them, and the variability of microelements and macroelements was minimal. However, the statistical results of our experiment do not support the stability of limiting elements hypothesis. We showed that there was no significant correlation between the variation coefficients of different elements and their actual detected contents and their potential physiological required contents. We speculate that active luxury absorption and passive adsorption may be the primary reasons for the departure from the “restrictive element stability hypothesis” (Patova et al., 2000; Patova and Sivkov, 2003).

### The Adaptability of *N. commune* to Regional Environment and the Response to Global Change

Higher plants have a relatively narrow geographical range compared to cyanobacteria, which has a higher dispersal ability and longer evolutionary age that contribute to cyanobacteria attaining a larger range (Slatyer et al., 2013). Cyanobacteria has evolved relatively perfect environmental adaptability, so that individual species have a wider geographic distribution. The evolution of Nostoc species has endured more extreme and complex climate changes than today and have survived successfully (Sand-Jensen and Jespersen, 2012). They not only have high genetic diversity (OTU > 97% related to the definition of species) (Walter et al., 2017), but also have a variety of physiological regulatory mechanisms (i.e., secretion of UV shielding pigment, secretion of large amounts of EPS, change of pigment composition ratio, and activation of antioxidant enzyme system) (Wang et al., 2013). Cyanobacteria, especially soil cyanobacteria *N. commune*, have enough tolerance capacity to be adapted to the current or future global changes.

Here, we found that the total carbon content did not increase significantly with increased drought and decreased temperature, which was different from previous observations in experimental control studies (Tamaru et al., 2005; Kehr and Dittmann, 2015; Chrismas et al., 2016). Therefore, we concluded that the increase of polysaccharides may be a short-term physiological adaptation, which overturns our traditional understanding. With increased drought and decreased temperature, there was a net increase of some mineral elements that led to an increase in ash content. Under drought and in low temperatures, *N. commune* can reduce the osmotic potential by increasing the mineral elements or the ash. Then it can rapidly absorb water when rainfall occurs, and rapidly grow and propagate under limited water resources. Therefore, the increase in mineral elements or ash may signal that *N. commune* has an adaptation mechanism for drought and low temperature environments, but this mechanism has not yet been reported.

## Conclusions

In this study, *N. commune* samples were collected across gradients of climate, soil, and atmospheric wet deposition mineral concentration in mainland China and fifteen minerals, including five macroelements (N, Ca, K, Fe, P), five microelements (Mn, Zn, Cu, Co, Se), and five heavy metals (Pb, Cr, As, Cd, Hg), were measured. Among the 15 elements investigated, 5 mineral elements showed significant geographical variation, while the others showed no significant geographical variation. Climatic factors could explain the variation of the geographically variable mineral elements (P, Cu, Zn, Co, Pb), which decreased with decreases in temperature and rainfall. Because there was no significant correlation between the variation coefficients of different elements and their actual detected contents and their potential physiological required contents, the soil cyanobacterial mineral elements did not follow the “restrictive element stability hypothesis” of higher plants.

## Data Availability Statement

The original contributions presented in the study are included in the article/Supplementary Material, further inquiries can be directed to the corresponding author/s.

## Author Contributions

All authors contributed intellectual input to this study and manuscript preparation. WW and QZ conceived the idea and designed the study. HL and XS conducted mineral analysis and collected the data with help from RG, YY, and XC. WW wrote the paper with input from all authors.

## Conflict of Interest

The authors declare that the research was conducted in the absence of any commercial or financial relationships that could be construed as a potential conflict of interest.
