# Supplementary material for: Geographical Variability of Mineral Elements and Stability of Restrictive Mineral Elements in Terrestrial Cyanobacteria Across Gradients of Climate, Soil, and Atmospheric Wet Deposition Mineral Concentration
Source: Front Microbiol. 2021 Jan 18;11:582655. doi: 10.3389/fmicb.2020.582655 (PMC7874062; doi:10.3389/fmicb.2020.582655)
Supplement: Supplementary file 1 [file Data_Sheet_1.docx]

**Supplementary information**

**Geographical variability of mineral elements and stability of restrictive mineral elements in terrestrial cyanobacteria across gradients of climate,** **soil and atmospheric wet deposition mineral concentration**

Weibo Wang, Hua Li, René Guénon, Yuyi Yang, Xiao Shu, Xiaoli Cheng, Quanfa Zhang

Table S1 Geographic characteristics of the sampling areas surveyed in this study.

| Sample areas | Latitude  (N/℃) | Longitude  (E/℃) | Altitude  (m) | Distance from  the equator  （km） | Distance from  the prime meridian  （km） | Sites |
| --- | --- | --- | --- | --- | --- | --- |
| 1 | 26.65 | 115.53 | 1253 | 2963.20 | 10921.77 | Xingguo County: Chongxian Town; Jun Cun Town; She Fu Town; Jiangbei Town; Chayuan Town. |
| 2 | 29.20 | 119.13 | 863 | 3246.74 | 10856.4 | Longyou County: Wucun Town; Xikou Town; Luojia Town. Changshan County: Tonggong Town; Baishi Town. |
| 3 | 34.20 | 108.75 | 695 | 3802.68 | 9394.19 | Changan District: Dayu Town; Weiziping Town. |
| 4 | 39.75 | 114.27 | 1920 | 4419.79 | 8945.18 | Yangyuan County: Dongjingji Town; Maquanbao Town. Guangling County: Nancun Town; Yixing Town. |
| 5 | 31.47 | 114.92 | 756 | 3499.14 | 10223.58 | Hongan County: Baliwan Town; Gaoqiao Town; Ercheng Town; Qiliming Town; Fangxihe Town. |
| 6 | 38.72 | 113.23 | 1500 | 4305.26 | 9040.29 | Xinfu District: Yangpo Town; Sanjiao Town; Zhuangmo Town.Yangqu County: Gaocun Town; Xilingjing Town. |
| 7 | 35.88 | 104.85 | 2025 | 3989.48 | 8884.82 | Huining County: Guochengyi Town; Hepan Town; Caijiamen Town; Xintianbao Town; Houjiachuan Town. |
| 8 | 29.95 | 118.52 | 1326 | 3330.13 | 10704.23 | Yixian County: Bishan Town; Longjiang Town, Yuting Town.Xiuning County: Landu Town. |
| 9 | 31.93 | 112.25 | 312 | 3550.28 | 9962.58 | Xiangcheng District: Wolong Town; Danyang Town; Oumiao Town. |
| 10 | 35.30 | 113.9 | 124 | 3924.99 | 9598.66 | Huixian County: Zhangcun Town; Tangzhuang Town; Changcun Town. Fengquan District: Luwangfen Town. |
| 11 | 29.52 | 118.97 | 521 | 3282.32 | 10798.94 | Chunan County: Fenkou Town; Longyuan Town; Fengshuling Town; Lishang Town; Fuxi Town. |
| 12 | 29.20 | 117.53 | 852 | 3246.74 | 10736.16 | Hengfeng County: Sipu Town. Guangxin County: dongtuan Town, Qingshui Town. Xinzhou County: Dawu Town. |
| 13 | 45.78 | 127.22 | 153 | 5090.26 | 8597.82 | Acheng District: Hongxing Town; Pingshan Town. Shangzhi County: Maoershan Town; Changshou Town. |
| 14 | 31.92 | 117.38 | 652 | 3549.17 | 10336.97 | Feixi County: Mingchuan Town; Guanting Town; Zipeng Town. |
| 15 | 36.63 | 101.8 | 2295 | 4072.87 | 8565.94 | Huangzhong District: Xibao Town; Huoshaogou village; Tianjiazai Town; Tuguanmen Town. |
| 16 | 37.87 | 105.22 | 1225 | 4210.75 | 8638.05 | Shapotou District: Yongkang Town; Xitai Town; Changle Town; Yingshuiqiao Town. |
| 17 | 34.68 | 110.68 | 1657 | 3856.06 | 9465.3 | Ruicheng County: Yangcheng Town; Dawang Town; Xuezhang Town; Donglu Town; Yangnan Town. |
| 18 | 28.20 | 114.9 | 542 | 3135.55 | 10668.9 | Yuanzhou District: Xicun Town; Tianping Town; Xinfang Town; Binjiang Town. Fenyi County: Fenyi Town. |
| 19 | 35.97 | 104.95 | 2140 | 3999.49 | 8879.84 | Anding District: Lijiabao Town; Qinglanshan Town; Xigongyi Town; Chankou Town. |
| 20 | 35.95 | 118.95 | 642 | 3997.27 | 9832.18 | Yinan County: Qingtuo Town, Fei County: Xuezhuang Town; Datianzhuang Town. Pingyi County: Bolin Town. |
| 21 | 40.93 | 114.43 | 1442 | 4550.99 | 8768.89 | Wanquan County: Wanquan Town; Beixintui Town; Beixintui Town; Gaomiaobao Town. |
| 22 | 30.70 | 111.25 | 546 | 3413.52 | 10053.65 | Dangyang County: Wangdian Town. Yiling District: Yaqueling Town; Longquan Town; Huanghua Town. |
| 23 | 34.95 | 108.37 | 597 | 3886.08 | 9263.56 | Eyi District: Shijing Town. Zhouzhi County: Jiufeng Town. |
| 24 | 34.53 | 113.72 | 256 | 3839.38 | 9699.31 | Xinmi County: Baizai Town. Yingyang County: Jiayu Town; Cimiao Town; Liuhe Town; Xiaoguan Town. |
| 25 | 44.28 | 116.72 | 1023 | 4923.47 | 8351.41 | Keshiketeng County: Xilamuren Riverbank. |
| 26 | 34.95 | 116.88 | 968 | 3886.08 | 9851.47 | Shanting District: Beizhuang Town; Xuzhuang Town; Shuiquan Town; Dianzi Town. |
| 27 | 32.95 | 112.68 | 652 | 3663.69 | 9852.11 | Zhenping County: Liuquanpu Town; Zheshan Town. Wolong District：Wangcun Town；Moshan Mountain. |
| 28 | 44.28 | 116.72 | 1023 | 4923.47 | 8351.41 | Xilinguole County：Yililete Town. Keshiketeng County: Xilamuren Riverbank. |
| 29 | 37.87 | 112.95 | 1542 | 4210.75 | 9151.77 | Wutai County：Donglei Town；Lingjing Town；Menxianshi Town； Gaohongmen Town; Chenjiazhuang Town. |
| 30 | 33.78 | 118.95 | 121 | 3755.98 | 10168.04 | Xuyi County: Guanzhong Town; Guantan Town. Shiyang County: Luji Town; Peiping Town. Hongze District: Xishunhe Town. |
| 31 | 29.47 | 110.33 | 1421 | 3276.76 | 10142.81 | Wulingyuan District: Xiehe Town; Xinqiao Town; Hezuoqiao Town. Cili County: Xujiafang Town; Ganyan Town. |
| 32 | 35.47 | 116.22 | 453.6 | 3943.89 | 9728.76 | Jiaxiang County: Tuanli Town; Jiaxiang Town; Maji Town; Zhifang Town. |
| 33 | 43.23 | 117.93 | 1256.3 | 4806.73 | 8590.88 | Alukeerqin County: Bayahua Town; Xianfeng Town; Shuangsheng Town. Balinzuo County: Longchang Town. |

Table S2 Biochemical contents of *N. commune* sampled from different areas in China.

| Sample areas | Protein (%) | Ash (%) | TOC (g kg^-1^) |
| --- | --- | --- | --- |
| 1 | 17.4 | 18.14 | 345.16 |
| 2 | 12.79 | 17.02 | 323.02 |
| 3 | 16.06 | 22.28 | 291.78 |
| 4 | 16.2 | 23.5 | 303.91 |
| 5 | 16.07 | 19.46 | 353.35 |
| 6 | 17.16 | 32.45 | 310.89 |
| 7 | 15 | 35.47 | 337.58 |
| 8 | 17.58 | 24.51 | 323.62 |
| 9 | 15.92 | 30.32 | 292.69 |
| 10 | 17.22 | 32.91 | 309.98 |
| 11 | 16.86 | 20.89 | 299.97 |
| 12 | 17.78 | 27.72 | 312.40 |
| 13 | 14.84 | 38.71 | 295.72 |
| 14 | 11.23 | 38.19 | 457.99 |
| 15 | 12.8 | 49.29 | 307.25 |
| 16 | 16.28 | 32.84 | 344.25 |
| 17 | 15.24 | 20.8 | 347.28 |
| 18 | 17.4 | 19.54 | 305.43 |
| 19 | 15.14 | 35.81 | 322.11 |
| 20 | 20.49 | 23.6 | 283.89 |
| 21 | 16.56 | 20.99 | 384.59 |
| 22 | 20.91 | NA | 307.25 |
| 23 | 15.97 | 21.08 | 295.72 |
| 24 | 16.63 | 25.74 | 303.91 |
| 25 | 14.72 | 33.05 | 262.36 |
| 26 | 16.26 | 26.36 | 349.41 |
| 27 | 16.67 | 14.9 | 289.05 |
| 28 | 17.41 | 19.95 | 289.65 |
| 29 | 16.17 | 23.62 | 319.38 |
| 30 | 18.5 | 19.54 | 251.74 |
| 31 | 16.78 | 25.19 | 304.82 |
| 32 | 28.19 | 20.23 | 368.51 |
| 33 | 17.46 | NA | 351.22 |

Table S3 Contents of 15 mineral elements in *N. commune* sampled from different areas in China.

| Sample areas | N  （g kg^-1^） | Ca  （g kg^-1^） | K  （g kg^-1^） | Fe  （g kg^-1^） | P  （g kg^-1^） | Mn  （mg kg^-1^） | Zn  （mg kg^-1^） | Cu  （mg kg^-1^） | Co  （mg kg^-1^） | Se  （mg kg^-1^） |
| --- | --- | --- | --- | --- | --- | --- | --- | --- | --- | --- |
| 1 | 23.84 | 10.45 | 3.63 | 2.93 | 0.789 | 107.74 | 29.64 | 7.66 | 0.46 | 0.260 |
| 2 | 23.44 | 4.44 | 2.64 | 1.84 | 0.538 | 76.00 | 22.25 | 5.60 | 0.70 | 0.223 |
| 3 | 26.14 | 5.22 | 3.32 | 1.70 | 0.678 | 93.23 | 26.71 | 6.98 | 1.79 | 0.189 |
| 4 | 23.27 | 4.82 | 3.79 | 1.11 | 1.322 | 96.43 | 22.65 | 6.74 | 2.56 | 0.187 |
| 5 | 25.85 | 4.23 | 2.87 | 2.36 | 0.567 | 74.55 | 19.50 | 5.99 | 1.01 | 0.173 |
| 6 | 30.16 | 10.14 | 4.19 | 3.76 | 0.978 | 191.50 | 49.06 | 11.04 | 3.30 | 0.264 |
| 7 | 28.27 | 6.28 | 4.03 | 3.47 | 0.644 | 137.68 | 30.94 | 9.43 | 4.23 | 0.259 |
| 8 | 30.85 | 11.35 | 2.83 | 2.49 | 0.571 | 104.16 | 30.03 | 7.36 | 1.20 | 0.207 |
| 9 | 27.00 | 10.84 | 4.59 | 3.85 | 1.230 | 159.30 | 42.55 | 10.86 | 3.60 | 0.263 |
| 10 | 30.56 | 9.54 | 5.78 | 3.94 | 0.789 | 163.35 | 43.69 | 11.61 | 4.11 | 0.261 |
| 11 | 25.97 | 10.56 | 3.38 | 2.99 | 0.576 | 120.89 | 29.41 | 8.31 | 1.59 | 0.191 |
| 12 | 29.42 | 9.13 | 3.69 | 3.35 | 0.726 | 138.20 | 30.69 | 8.65 | 2.14 | 0.204 |
| 13 | 21.54 | 11.38 | 5.89 | 1.28 | 0.625 | 167.00 | 37.70 | 10.84 | 4.41 | 0.267 |
| 14 | 28.44 | 9.60 | 6.36 | 2.11 | 0.581 | 201.79 | 34.43 | 10.10 | 3.90 | 0.163 |
| 15 | 21.66 | 12.60 | 7.29 | 1.90 | 0.630 | 185.88 | 55.98 | 13.93 | 5.69 | 0.255 |
| 16 | 24.99 | 10.04 | 4.59 | 2.10 | 0.809 | 155.94 | 38.15 | 10.33 | 3.64 | 0.165 |
| 17 | 24.88 | 6.64 | 3.19 | 0.82 | 0.562 | 113.75 | 27.80 | 7.18 | 2.66 | 0.188 |
| 18 | 23.84 | 9.76 | 3.17 | 0.91 | 0.596 | 117.68 | 26.61 | 7.66 | 1.64 | 0.145 |
| 19 | 22.81 | 10.57 | 4.86 | 1.24 | 0.630 | 175.63 | 37.69 | 10.94 | 3.80 | 0.213 |
| 20 | 29.01 | 11.17 | 3.51 | 0.95 | 0.630 | 117.46 | 70.39 | 11.56 | 2.84 | 0.199 |
| 21 | 22.98 | 3.39 | 2.74 | 0.84 | 1.666 | 98.95 | 25.15 | 7.43 | 2.44 | 0.092 |
| 22 | 31.31 | 8.85 | 3.18 | 0.90 | 0.823 | 165.96 | 28.36 | 6.71 | 1.63 | 0.163 |
| 23 | 23.27 | 3.69 | 2.68 | 1.01 | 0.499 | 94.29 | 22.78 | 7.18 | 2.14 | 0.062 |
| 24 | 21.54 | 8.96 | 3.74 | 1.18 | 0.688 | 166.16 | 36.38 | 9.88 | 3.59 | 0.140 |
| 25 | 22.00 | 10.94 | 5.17 | 1.33 | 0.939 | 171.73 | 42.94 | 11.56 | 5.11 | 0.256 |
| 26 | 26.72 | 10.42 | 4.49 | 1.52 | 0.615 | 142.40 | 29.54 | 8.98 | 3.21 | 0.185 |
| 27 | 28.15 | 10.40 | 4.48 | 1.51 | 0.615 | 142.01 | 29.81 | 9.03 | 3.14 | 0.165 |
| 28 | 26.14 | 10.06 | 2.94 | 0.58 | 0.620 | 117.24 | 32.48 | 9.15 | 2.86 | 0.192 |
| 29 | 21.26 | 3.70 | 3.33 | 1.08 | 0.552 | 95.04 | 25.95 | 6.88 | 3.41 | 0.232 |
| 30 | 25.57 | 9.78 | 2.21 | 2.40 | 0.644 | 121.63 | 29.96 | 8.55 | 2.35 | 0.242 |
| 31 | 19.65 | 4.50 | 3.63 | 1.01 | 0.644 | 94.71 | 26.45 | 7.41 | 4.24 | 0.193 |
| 32 | 46.65 | 9.90 | 3.35 | 0.83 | 1.826 | 77.46 | 67.35 | 12.73 | 3.29 | 0.184 |
| 33 | 27.29 | 7.96 | 4.04 | 1.79 | 1.182 | 154.09 | 42.53 | 12.61 | 4.43 | 0.229 |

Table S3 continued Contents of 15 mineral elements in *N. commune* sampled from different areas in China.

| Sample areas | Pb  （g kg^-1^） | Cr  （g kg^-1^） | As  （g kg^-1^） | Cd  （g kg^-1^） | Hg  （g kg^-1^） |
| --- | --- | --- | --- | --- | --- |
| 1 | 10.21 | 0.423 | 2.48 | 0.423 | 0.023 |
| 2 | 8.76 | 0.325 | 2.09 | 0.325 | 0.025 |
| 3 | 11.29 | 0.428 | 2.36 | 0.428 | 0.020 |
| 4 | 10.33 | 0.423 | 2.99 | 0.423 | 0.018 |
| 5 | 10.20 | 0.430 | 3.16 | 0.430 | 0.019 |
| 6 | 27.36 | 0.483 | 4.72 | 0.483 | 0.021 |
| 7 | 14.35 | 0.460 | 4.67 | 0.460 | 0.022 |
| 8 | 10.11 | 0.418 | 2.99 | 0.418 | 0.020 |
| 9 | 17.65 | 0.523 | 5.59 | 0.523 | 0.023 |
| 10 | 19.50 | 0.595 | 4.79 | 0.595 | 0.022 |
| 11 | 14.10 | 0.370 | 4.21 | 0.370 | 0.015 |
| 12 | 11.89 | 0.285 | 5.37 | 0.285 | 0.016 |
| 13 | 17.53 | 0.450 | 6.91 | 0.450 | 0.020 |
| 14 | 16.18 | 0.388 | 5.13 | 0.388 | 0.015 |
| 15 | 23.70 | 0.538 | 8.05 | 0.538 | 0.018 |
| 16 | 17.83 | 0.370 | 6.81 | 0.370 | 0.022 |
| 17 | 11.71 | 0.293 | 4.69 | 0.293 | 0.016 |
| 18 | 11.11 | 0.343 | 3.87 | 0.343 | 0.018 |
| 19 | 16.39 | 0.313 | 5.33 | 0.313 | 0.021 |
| 20 | 37.59 | 0.420 | 3.57 | 0.420 | 0.035 |
| 21 | 12.81 | 0.298 | 3.40 | 0.298 | 0.017 |
| 22 | 9.65 | 0.268 | 1.63 | 0.268 | 0.017 |
| 23 | 10.55 | 0.250 | 2.72 | 0.250 | 0.017 |
| 24 | 15.70 | 0.365 | 3.40 | 0.365 | 0.018 |
| 25 | 20.70 | 0.488 | 5.30 | 0.488 | 0.018 |
| 26 | 13.09 | 0.388 | 3.43 | 0.388 | 0.018 |
| 27 | 13.91 | 0.335 | 3.57 | 0.335 | 0.018 |
| 28 | 14.13 | 0.403 | 2.99 | 0.403 | 0.016 |
| 29 | 12.53 | 0.348 | 2.60 | 0.348 | 0.015 |
| 30 | 15.06 | 0.458 | 3.50 | 0.458 | 0.015 |
| 31 | 14.33 | 0.438 | 3.70 | 0.438 | 0.081 |
| 32 | 27.54 | 0.460 | 3.04 | 0.460 | 0.023 |
| 33 | 19.34 | 0.383 | 3.28 | 0.383 | 0.034 |

Table S4 Climate characteristics of the sampling areas surveyed in this study.

| Sample areas | Mean annual temperature (MAT, ℃) | Mean annual precipitation  (MAP, mm) | Mean annual solar radiation  (MAR, MJ m^-2^ day^-1^) | Mean maximum annual temperature (MAT_max_, ℃) | Mean minimum annual temperature (MAT_min_, ℃) | Mean annual precipitation  days (MAPD, d) | Relative humidity  (RH, %) | Frost free  days  (FFD, d) | Mean annual  evaporation  (MAE, mm) | Drought  index  (DI) | UV  radiation  (MJ m^-2^ year^-1^) |
| --- | --- | --- | --- | --- | --- | --- | --- | --- | --- | --- | --- |
| 1 | 18.8 | 1515.6 | 458.4 | 24.76 | 16.47 | 112.3 | 76 | 284 | 1635.8 | 1.08 | 200.5 |
| 2 | 16.9 | 1843 | 456 | 22.80 | 14.23 | 159.5 | 78.8 | 255 | 1392 | 0.76 | 200.5 |
| 3 | 13.6 | 561.2 | 457 | 20.30 | 10.92 | 89.1 | 68.2 | 221 | 1134.7 | 2.02 | 193.3 |
| 4 | 7.1 | 408.8 | 561.7 | 14.81 | 1.82 | 73.9 | 51.4 | 130 | 1962 | 4.80 | 165.1 |
| 5 | 17.6 | 1023.3 | 474 | 22.20 | 14.79 | 147 | 77 | 246 | 1443.3 | 1.41 | 180.4 |
| 6 | 9.3 | 405 | 595.6 | 16.92 | 4.67 | 70.3 | 61 | 160 | 1561 | 3.85 | 165.1 |
| 7 | 7.9 | 332.6 | 595.6 | 15.26 | 2.64 | 96 | 56 | 155 | 1800 | 5.41 | 244.9 |
| 8 | 16.3 | 1670 | 480 | 12.06 | 6.06 | 158.8 | 78 | 237 | 1679.8 | 1.01 | 200.5 |
| 9 | 16.2 | 878.3 | 422 | 21.48 | 12.62 | 117.7 | 76.1 | 241 | 1434 | 1.63 | 180.4 |
| 10 | 14 | 656.3 | 472 | 20.29 | 10.45 | 75.5 | 68 | 220 | 1748.4 | 2.66 | 180.3 |
| 11 | 19.1 | 1319.7 | 450 | 22.25 | 14.35 | 155 | 76 | 245 | 1355.1 | 1.03 | 200.5 |
| 12 | 17.1 | 1750 | 462 | 23.31 | 14.51 | 178 | 76 | 265 | 1382 | 0.79 | 200.5 |
| 13 | 3.6 | 569.1 | 472 | 10.79 | 0.41 | 104.4 | 65.4 | 136 | 1586.8 | 2.79 | 181.9 |
| 14 | 15.7 | 1074.3 | 497.9 | 21.22 | 13.13 | 113.8 | 75.8 | 234 | 862.6 | 0.80 | 172.5 |
| 15 | 7.6 | 380.1 | 576 | 14.60 | -0.23 | 96.9 | 55.2 | 142 | 1363.6 | 3.59 | 261.4 |
| 16 | 8.6 | 213.3 | 597 | 17.24 | 3.57 | 51.6 | 57 | 163 | 1729.6 | 8.11 | 228.8 |
| 17 | 11.3 | 533.8 | 542 | 20.16 | 10.25 | 82.2 | 66 | 173 | 1858.5 | 3.48 | 165.1 |
| 18 | 17.3 | 1720 | 438.9 | 23.05 | 15.60 | 174 | 81 | 267 | 1339 | 0.78 | 200.5 |
| 19 | 7 | 450 | 589 | 15.26 | 2.64 | 105 | 68.1 | 150 | 1500 | 3.33 | 244.9 |
| 20 | 14.1 | 849 | 497 | 19.51 | 9.90 | 82.6 | 69 | 213 | 1128 | 1.33 | 182.6 |
| 21 | 7.8 | 349.7 | 576 | 16.02 | 4.57 | 75.3 | 60 | 127 | 1400 | 4.00 | 228.2 |
| 22 | 16.8 | 1236.3 | 420 | 22.15 | 14.09 | 135.4 | 75 | 261 | 1271 | 1.03 | 180.4 |
| 23 | 12.5 | 634.2 | 480 | 20.30 | 10.92 | 89.7 | 70 | 215 | 1064 | 1.68 | 193.3 |
| 24 | 14.1 | 640.9 | 528.5 | 20.68 | 10.82 | 79.3 | 65.2 | 220 | 1476 | 2.30 | 180.3 |
| 25 | 1.7 | 295 | 599 | 10.74 | -2.88 | 78.6 | 57.4 | 142 | 1747 | 5.92 | 228.2 |
| 26 | 12.8 | 998 | 497 | 19.67 | 10.00 | 65 | 66 | 196 | 1678 | 1.68 | 182.6 |
| 27 | 14.6 | 729.3 | 453 | 20.74 | 11.43 | 95.4 | 75 | 235 | 1298 | 1.78 | 180.3 |
| 28 | 1.7 | 295 | 599 | 10.74 | -2.88 | 78.6 | 57.4 | 142 | 1747 | 5.92 | 228.2 |
| 29 | 9.8 | 452 | 564.3 | 6.79 | -1.56 | 70.3 | 56 | 158 | 1718 | 3.80 | 165.1 |
| 30 | 14.5 | 1724.6 | 481 | 19.93 | 11.12 | 104 | 79 | 224 | 1385 | 0.80 | 172.5 |
| 31 | 17.2 | 1401 | 390 | 21.62 | 13.13 | 165 | 78 | 250 | 1140 | 0.81 | 184.1 |
| 32 | 13.7 | 763 | 496 | 20.07 | 9.05 | 73 | 68 | 201 | 1694 | 2.22 | 182.6 |
| 33 | 2.3 | 371 | 511.6 | 15.24 | 1.79 | 70.1 | 48.8 | 156 | 2067 | 5.57 | 223.9 |

Table S5 The background values of soil and plant mineral elements in China.

†Soil data were obtained from the national soil survey, ‡ plant data were obtained from Han *et al.*, (2011).

|  | Fe | K | Ca | Al | Na | Mg | Mn | Se | Zn | Cr | Pb | Cu |
| --- | --- | --- | --- | --- | --- | --- | --- | --- | --- | --- | --- | --- |
| Soil element† (mg kg^-1^) | 29400 | 18600 | 15400 | 6620 | 1020 | 780 | 583 | 167 | 74.2 | 61 | 26 | 22.6 |
| Plant element ‡ (mg g^-1^) | 0.49 | 13.1 | 13.7 | 0.95 | 7.28 | 3.13 | 0.43 | 7.47 |  |  |  |  |
|  | Co | As | Cd | Hg | Co |  |  |  |  |  |  |  |
| Soil element† (mg kg^-1^) | 12.7 | 11.2 | 0.097 | 0.065 | 12.7 |  |  |  |  |  |  |  |
| Plant element ‡ (mg g^-1^) |  |  |  |  |  |  |  |  |  |  |  |  |

Table S6 Edaphic characteristics of the sampling areas surveyed in this study.

† Data were obtained from the national soil survey, ‡ data were from soil pollution condition investigation communiqué, N/A stands for not available.

| Study  areas | pH† | TN†  (%) | TP†  (%) | TK†  (%) | Cd‡  (mg/kg) | Cr‡  (mg/kg) | As‡  (mg/kg) | Hg‡  (mg/kg) | Pb‡  (mg/kg) | Cu‡  (mg/kg) | Zn‡  (mg/kg) | TOC†  (%) | C/N† | N/P† |
| --- | --- | --- | --- | --- | --- | --- | --- | --- | --- | --- | --- | --- | --- | --- |
| 1 | 4.87 | 0.44 | 0.29 | 23.6 | 0.187 | 65.4 | 20.9 | 0.109 | 38 | 32.1 | 93.3 | 7.65 | 10.11 | 1.52 |
| 2 | 6.5 | 1.1 | 0.39 | N/A | 0.211 | 51.1 | 9 | 0.139 | 40 | 26.2 | 95.9 | 19.78 | 10.45 | 2.82 |
| 3 | 8.12 | 0.77 | 0.66 | 18.12 | 0.266 | 79 | 12.2 | 0.1 | 29.2 | 29.6 | 81.8 | 12.28 | 9.27 | 1.17 |
| 4 | 8.19 | 0.43 | 0.6 | N/A | 0.154 | 63.5 | 9.5 | 0.045 | 19.6 | 23.4 | 64.2 | 6.6 | 8.92 | 0.72 |
| 5 | 6.55 | 0.8 | 0.64 | 18.65 | 0.313 | 70.6 | 13 | 0.083 | 29.3 | 29.4 | 72.4 | 14.85 | 10.79 | 1.25 |
| 6 | 8.4 | 0.48 | 0.7 | N/A | 0.154 | 63.5 | 9.5 | 0.045 | 19.6 | 23.4 | 64.2 | 6.43 | 7.79 | 0.69 |
| 7 | 8.5 | 0.65 | 0.63 | 18.7 | 0.146 | 68.2 | 12.2 | 0.04 | 24.5 | 25.2 | 71.8 | 9.85 | 8.81 | 1.03 |
| 8 | 7.38 | 0.95 | 0.5 | 20.08 | 0.161 | 69.1 | 10 | 0.059 | 26.7 | 26.4 | 65.4 | 15.7 | 9.61 | 1.90 |
| 9 | 7.75 | 0.88 | 0.64 | 19.22 | 0.313 | 70.6 | 13 | 0.083 | 29.3 | 29.4 | 72.4 | 13.75 | 9.08 | 1.38 |
| 10 | 8.22 | 0.8 | 0.6 | 18.5 | 0.18 | 74.3 | 10.7 | 0.046 | 26.8 | 21.8 | 63.7 | 12.7 | 9.23 | 1.33 |
| 11 | 7.5 | 2.2 | 0.69 | 12.5 | 0.211 | 51.1 | 9 | 0.139 | 40 | 26.2 | 95.9 | 35.05 | 9.26 | 3.19 |
| 12 | 5.83 | 1.41 | 0.47 | 14.28 | 0.187 | 65.4 | 20.9 | 0.109 | 38 | 32.1 | 93.3 | 27.45 | 11.32 | 3.00 |
| 13 | 6.1 | 1.91 | 0.65 | 29.9 | 0.109 | 60.8 | 8.5 | 0.05 | 21.6 | 21.1 | 65.3 | 38.4 | 11.69 | 2.94 |
| 14 | 6.99 | 0.95 | 0.34 | 15.49 | 0.161 | 69.1 | 10 | 0.059 | 26.7 | 26.4 | 65.4 | 15.86 | 9.71 | 2.79 |
| 15 | 8.61 | 1.12 | 0.74 | 18.69 | 0.172 | 62.4 | 14.5 | 0.03 | 22.5 | 21.7 | 66.9 | 19.63 | 10.19 | 1.51 |
| 16 | 8.46 | 0.58 | 0.64 | 19.21 | 0.136 | 66 | 11.6 | 0.025 | 19 | 22.9 | 58.6 | 8.1 | 8.12 | 0.91 |
| 17 | 8.19 | 0.68 | 0.59 | N/A | 0.154 | 63.5 | 9.5 | 0.045 | 19.6 | 23.4 | 64.2 | 8.8 | 7.52 | 1.15 |
| 18 | 5.42 | 1.79 | 0.48 | 16.44 | 0.187 | 65.4 | 20.9 | 0.109 | 38 | 32.1 | 93.3 | 32.14 | 10.44 | 3.73 |
| 19 | 8.41 | 0.85 | 0.66 | 19.03 | 0.146 | 68.2 | 12.2 | 0.04 | 24.5 | 25.2 | 71.8 | 12.13 | 8.30 | 1.29 |
| 20 | 7.16 | 0.57 | 0.34 | 17.42 | 0.121 | 60.4 | 9.1 | 0.037 | 24.2 | 24.8 | 73.1 | 8.56 | 8.73 | 1.68 |
| 21 | 8.38 | 0.73 | 0.73 | 15.71 | 0.159 | 61.4 | 9.2 | 0.054 | 22 | 23 | 68.7 | 9.89 | 7.88 | 1.00 |
| 22 | 6.57 | 1.03 | 0.42 | 17.86 | 0.313 | 70.6 | 13 | 0.083 | 29.3 | 29.4 | 72.4 | 16.2 | 9.14 | 2.45 |
| 23 | 8.33 | 0.84 | 0.58 | 19.57 | 0.266 | 79 | 12.2 | 0.1 | 29.2 | 29.6 | 81.8 | 13.06 | 9.04 | 1.45 |
| 24 | 8.34 | 0.76 | 0.57 | 17.68 | 0.18 | 74.3 | 10.7 | 0.046 | 26.8 | 21.8 | 63.7 | 12.26 | 9.38 | 1.33 |
| 25 | 7.69 | 1.51 | 0.72 | 28.03 | 0.086 | 45.7 | 7.7 | 0.031 | 21.6 | 15.2 | 55.4 | 28.43 | 10.95 | 2.10 |
| 26 | 7.31 | 0.61 | 0.39 | 18.78 | 0.121 | 60.4 | 9.1 | 0.037 | 24.2 | 24.8 | 73.1 | 9.62 | 9.17 | 1.56 |
| 27 | 7.53 | 0.82 | 0.49 | 19.17 | 0.18 | 74.3 | 10.7 | 0.046 | 26.8 | 21.8 | 63.7 | 13.36 | 9.47 | 1.67 |
| 28 | 7.69 | 1.51 | 0.72 | 28.03 | 0.086 | 45.7 | 7.7 | 0.031 | 21.6 | 15.2 | 55.4 | 28.43 | 10.95 | 2.10 |
| 29 | 8.4 | 0.97 | 0.55 | N/A | 0.154 | 63.5 | 9.5 | 0.045 | 19.6 | 23.4 | 64.2 | 22.7 | 13.61 | 1.76 |
| 30 | 8.35 | 0.69 | 0.65 | 18.19 | 0.177 | 80.1 | 9.7 | 0.133 | 33.1 | 27.7 | 78 | 9.4 | 7.92 | 1.06 |
| 31 | 6.17 | 1.73 | 0.46 | 17.58 | 0.299 | 62.5 | 15.9 | 0.14 | 40.5 | 28.8 | 102.9 | 38.52 | 12.95 | 3.76 |
| 32 | 8 | 0.37 | 0.57 | 16.5 | 0.121 | 60.4 | 9.1 | 0.037 | 24.2 | 24.8 | 73.1 | 5.3 | 8.33 | 0.65 |
| 33 | 8.16 | 1.03 | 0.78 | 21.74 | 0.086 | 45.7 | 7.7 | 0.031 | 21.6 | 15.2 | 55.4 | 15.5 | 8.75 | 1.32 |

Table S7 Referenced atmospheric total deposition flux and wet deposition flux of mineral elements in this study.

†Precipitation and dry-deposited particles were collected simultaneously at 10 sites in Northern China from December 2007 to November 2010, and the data were from Pan and Wang (2015); ‡long-term mean values of the daily wet deposition of trace elements measured from April 1987 to March 1991 at the site “Postturm”, 40 km east-northeast of the city of Hamburg, obtained by Michaelis (1997).

| Mineral elements | Total deposition†  (mg/m ^2^.year) | Wet deposition‡  (μg/m^2^ d) |
| --- | --- | --- |
| K | 1759.6 | 210 |
| Ca | 8334 | 660 |
| Cr | 7.28 | 1.3 |
| Mn | 85.1 | 11 |
| Fe | 3957.1 | 180 |
| Cu | 15.1 | 4.4 |
| Zn | 106.5 | 53 |
| As | 4.25 | 1.1 |
| Se | 1.9 | 0.5 |
| Cd | 0.54 | 0.6 |
| Pb | 26.5 | 11 |
| Co | 3.45 | NA |

Table S8 Atmospheric wet deposition concentrations of the sampling areas surveyed in this study.

† Data were obtained from the observation by Xie and Li (2012), ‡ data were obtained from the Kriging interpolation by the software arcGIS 10.

| Study  areas | pH‡ | Ca†  (μmol /L) | K†  (μmol /L) | N‡  (μg/L) | P‡  (μg/L) | Cr‡  (μg/L) | Pb‡  (μg/L) | Cd‡  (μg/L) | Salinity  (mg/L) |
| --- | --- | --- | --- | --- | --- | --- | --- | --- | --- |
| 1 | 6.07 | 137.7 | 11.11 | 203.32 | 52.62 | 8.12 | 2.23 | 5.10 | 77.18 |
| 2 | 6.28 | 83.55 | 13.41 | 204.58 | 83.62 | 6.93 | 1.76 | 3.38 | 125.71 |
| 3 | 7.96 | 416 | 23.55 | 580.21 | 40.06 | 17.64 | 2.99 | 5.70 | 91.25 |
| 4 | 6.07 | 196.1 | 9.9 | 964.95 | 28.14 | 30.51 | 6.96 | 5.07 | 98.81 |
| 5 | 5.82 | 137.7 | 11.11 | 343.30 | 65.18 | 11.80 | 2.89 | 8.67 | 96.33 |
| 6 | 6.11 | 196.1 | 9.9 | 1002.64 | 36.99 | 29.11 | 5.60 | 7.21 | 100.59 |
| 7 | 7.30 | 416 | 23.55 | 1029.39 | 116.30 | 20.28 | 4.68 | 3.77 | 97.66 |
| 8 | 6.26 | 83.55 | 13.41 | 217.21 | 88.28 | 7.45 | 1.97 | 3.84 | 105.48 |
| 9 | 6.04 | 137.7 | 11.11 | 387.80 | 23.54 | 13.08 | 2.94 | 9.32 | 88.64 |
| 10 | 7.04 | 137.7 | 11.11 | 852.00 | 39.75 | 17.20 | 2.70 | 8.19 | 121.56 |
| 11 | 6.29 | 83.55 | 13.41 | 286.11 | 116.43 | 9.67 | 2.45 | 4.73 | 125.18 |
| 12 | 5.89 | 137.7 | 11.11 | 203.69 | 71.31 | 7.19 | 1.93 | 4.01 | 90.50 |
| 13 | 6.66 | 457.4 | 47.95 | 383.39 | 39.30 | 13.61 | 1.77 | 2.44 | 61.42 |
| 14 | 6.50 | 83.55 | 13.41 | 337.51 | 114.61 | 10.89 | 2.60 | 6.55 | 107.86 |
| 15 | 6.81 | 416 | 23.55 | 847.07 | 116.23 | 18.88 | 4.38 | 3.82 | 101.05 |
| 16 | 6.80 | 416 | 23.55 | 1994.74 | 136.58 | 22.05 | 6.93 | 4.81 | 109.68 |
| 17 | 7.30 | 196.1 | 9.9 | 662.45 | 12.58 | 20.34 | 3.36 | 8.46 | 101.39 |
| 18 | 5.40 | 137.7 | 11.11 | 185.42 | 41.05 | 7.25 | 2.13 | 4.82 | 86.03 |
| 19 | 7.29 | 416 | 23.55 | 758.46 | 84.38 | 14.77 | 3.44 | 2.69 | 97.90 |
| 20 | 6.97 | 83.55 | 13.41 | 449.27 | 135.00 | 13.09 | 3.38 | 3.74 | 116.07 |
| 21 | 6.17 | 196.1 | 9.9 | 1135.12 | 29.62 | 36.97 | 7.40 | 6.06 | 93.97 |
| 22 | 5.88 | 137.7 | 11.11 | 236.22 | 18.82 | 9.89 | 2.61 | 6.63 | 72.20 |
| 23 | 8.13 | 416 | 23.55 | 500.16 | 36.41 | 14.34 | 2.07 | 3.66 | 93.72 |
| 24 | 7.03 | 137.7 | 11.11 | 778.70 | 43.54 | 17.83 | 3.10 | 9.93 | 119.70 |
| 25 | 6.55 | 196.1 | 9.9 | 978.34 | 69.37 | 36.31 | 3.68 | 5.96 | 79.13 |
| 26 | 7.13 | 83.55 | 13.41 | 443.94 | 86.54 | 11.55 | 2.73 | 5.08 | 122.71 |
| 27 | 6.49 | 137.7 | 11.11 | 512.26 | 29.27 | 15.34 | 3.35 | 10.80 | 101.92 |
| 28 | 6.52 | 196.1 | 9.9 | 978.34 | 69.42 | 36.32 | 3.89 | 6.03 | 77.95 |
| 29 | 6.24 | 196.1 | 9.9 | 922.62 | 34.52 | 25.63 | 4.40 | 7.21 | 106.14 |
| 30 | 6.96 | 83.55 | 13.41 | 225.03 | 82.60 | 6.54 | 1.49 | 2.89 | 115.31 |
| 31 | 6.11 | 137.7 | 11.11 | 206.75 | 23.38 | 8.69 | 2.27 | 5.49 | 78.46 |
| 32 | 7.14 | 83.55 | 13.41 | 657.61 | 88.24 | 14.81 | 3.44 | 6.10 | 124.33 |
| 33 | 6.76 | 196.1 | 9.9 | 836.20 | 74.26 | 32.19 | 3.46 | 5.25 | 78.66 |

Table S9 Descriptive statistics of biochemical compositions of minerals in *N. commune* sampled from different areas in China.

|  | Mean value | Maximum value | Minimum  value | Standard deviation | Coefficient of variation (%) |
| --- | --- | --- | --- | --- | --- |
| Protein (%) (n = 32) | 16.35 | 20.91 | 11.23 | 1.92 | 11.74 |
| Ash (%) (n = 29) | 26.46 | 49.29 | 14.9 | 7.89 | 29.82 |
| TOC(g kg^-1^) (n = 33) | 319.57 | 457.99 | 251.74 | 38.17 | 11.95 |
| N (g kg^-1^) (n = 33) | 26.20 | 46.65 | 19.96 | 4.82 | 18.42 |
| P (g kg^-1^) (n = 33) | 0.78 | 1.83 | 0.50 | 0.32 | 0.41 |
| K (g kg^-1^) (n = 33) | 3.98 | 7.29 | 2.21 | 1.16 | 0.30 |
| Cu(mgkg^-1^) (n = 33) | 9.12 | 13.93 | 5.6 | 2.15 | 23.57 |
| Zn(mgkg^-1^) (n = 33) | 34.71 | 70.39 | 19.5 | 12.01 | 34.60 |
| Fe(mgkg^-1^) (n = 33) | 1851.32 | 3943.63 | 582.88 | 1000.62 | 54.04 |
| Mn(mgkg^-1^) (n = 33) | 131.51 | 201.79 | 74.55 | 35.97 | 27.35 |
| Co(mgkg^-1^) (n = 33) | 2.94 | 5.69 | 0.46 | 1.27 | 43.20 |
| Se(mgkg^-1^) (n = 33) | 0.2 | 0.27 | 0.06 | 0.05 | 0.25 |
| Ca(mgkg^-1^) (n = 33) | 8525.01 | 12600.00 | 3390.6 | 2755.4 | 32.32 |
| Pb(mgkg^-1^) (n = 33) | 15.66 | 37.59 | 8.76 | 6.19 | 39.53 |
| Cd(mgkg^-1^) (n = 33) | 0.40 | 0.60 | 0.25 | 0.08 | 20.00 |
| Cr(mgkg^-1^) (n = 33) | 14.74 | 33.69 | 6.7 | 5.78 | 39.21 |
| Hg(mgkg^-1^) (n = 33) | 0.022 | 0.081 | 0.015 | 0.012 | 54.54 |
| As(mgkg^-1^) (n = 33) | 4.01 | 8.05 | 1.63 | 1.47 | 36.66 |

Table S10 Correlations between *N. commune* biochemicals and minerals with geographic variables. Levels of statistical significance: **p* < 0.05, ***p* < 0.01 and ****p* < 0.001.

| biochemicals and minerals  (mg g^-1^protein) | Distance from the equator (10^3^ km) | | | | Distance from the prime meridian (10^3^ km) | | | |
| --- | --- | --- | --- | --- | --- | --- | --- | --- |
|  | Pearson correlation | | Spearman’s rank correlation | | Pearson correlation | | Spearman’s rank correlation | |
|  | *r* | *p* | *ρ* | *p* | *r* | *p* | *ρ* | *p* |
| Ash | 0.269 | 0.144 | 0.349 | 0.054 | **-0.391** | **0.030*** | **-0.430** | **0.016*** |
| TOC | -0.055 | 0.777 | 0.115 | 0.524 | 0.013 | 0.944 | -0.169 | 0.347 |
| N | -0.108 | 0.550 | -0.083 | 0.644 | 0.112 | 0.534 | 0.083 | 0.646 |
| Ca | 0.034 | 0.852 | 0.064 | 0.724 | 0.000 | 1.000 | -0.055 | 0.762 |
| K | 0.232 | 0.193 | 0.289 | 0.103 | -0.322 | 0.067 | **-0.356** | **0.042*** |
| Fe | -0.277 | 0.119 | -0.250 | 0.161 | 0.241 | 0.176 | 0.251 | 0.159 |
| P | **0.399** | **0.021*** | **0.375** | **0.031*** | **-0.390** | **0.025*** | **-0.385** | **0.027*** |
| Mn | 0.232 | 0.194 | 0.294 | 0.097 | -0.302 | 0.087 | -0.357 | 0.042* |
| Zn | **0.355** | **0.043*** | **0.411** | **0.018*** | **-0.372** | **0.033*** | **-0.371** | **0.034*** |
| Cu | **0.423** | **0.014*** | **0.456** | **0.008**** | **-0.468** | **0.006**** | **-0.469** | **0.006**** |
| Co | **0.592** | **0.000***** | **0.582** | **0.000***** | **-0.675** | **0.000***** | **-0.643** | **0.000***** |
| Se | 0.098 | 0.589 | 0.158 | 0.379 | -0.081 | 0.654 | -0.158 | 0.380 |
| Pb | **0.457** | **0.008**** | **0.511** | **0.002**** | **-0.458** | **0.007**** | **-0.468** | **0.006**** |
| Cr | 0.157 | 0.383 | 0.101 | 0.577 | -0.276 | 0.120 | -0.198 | 0.269 |
| As | 0.249 | 0.162 | 0.226 | 0.206 | -0.327 | 0.063 | -0.296 | 0.095 |
| Cd | 0.195 | 0.276 | 0.149 | 0.406 | -0.213 | 0.235 | -0.151 | 0.402 |
| Hg | -0.048 | 0.793 | 0.119 | 0.509 | -0.034 | 0.849 | -0.180 | 0.316 |

Table S11 Linear regressions of *N. commune* minerals on MAT and MAP.

|  |  | Model(*r^2^*) | | |
| --- | --- | --- | --- | --- |
| Mineral | N | 1 MAP | 2 MAT | 3 MAP+MAT |
| P | 33 | 0.164 | 0.144 |  |
| K | 33 | 0.125 |  |  |
| Zn | 33 | 0.147 | 0.132 |  |
| Cu | 33 | 0.176 | 0.232 | 0.245 |
| Co | 33 | 0.403 | 0.366 | 0.432 |
| Pb | 33 | 0.225 | 0.175 | 0.230 |

Table S12 Model summary for the stepwise multiple regression of *N. commune* minerals on five climatic variables (MAP, MAT, MAR, MAT_max_, MAT_min_).

| Mineral | Adj. r^2^  Full model | Contribution of the individual predictor | | | | |
| --- | --- | --- | --- | --- | --- | --- |
|  |  | MAP | MAT | MAR | MAT_max_ | MAT_min_ |
| P | 0.137 | 100 |  |  |  |  |
| K | 0.097 | 100 |  |  |  |  |
| Zn | 0.119 | 100 |  |  |  |  |
| Cu | 0.207 |  | 100 |  |  |  |
| Co | 0.384 | 100 |  |  |  |  |
| Pb | 0.200 | 100 |  |  |  |  |

Table S13 Spearman’s rank correlations (*ρ*) between Nostoc minerals and soil pH, and between atmospheric wet deposition pH and atmospheric wet deposition total salinity. Nostoc minerals (mg g^-1^protein) and atmospheric wet deposition total salinity (mg l^-1^) are log_10_-tranformed before analysis.

| N=33 | N | Ca | K | Fe | P | Mn | Zn | Cu | Co | Se | Pb | Cr | As | Cd | Hg |
| --- | --- | --- | --- | --- | --- | --- | --- | --- | --- | --- | --- | --- | --- | --- | --- |
| soil pH | 0.103  0.569 | -0.083  0.644 | 0.180  0.315 | 0.065  0.721 | 0.260  0.144 | 0.265  0.136 | 0.234  0.191 | 0.310  0.079 | 0.433  0.012* | 0.110  0.541 | 0.358  0.041* | 0.214  0.231 | 0.207  0.248 | 0.092  0.611 | 0.014  0.940 |
| Rainfall pH | 0.158  0.380 | 0.046  0.800 | 0.156  0.387 | -0.109  0.547 | 0.048  0.789 | 0.113  0.531 | 0.374  0.032* | 0.382  0.028* | 0.338  0.054 | 0.034  0.852 | 0.356  0.042* | 0.078  0.667 | 0.097  0.593 | 0.006  0.972 | 0.108  0.549 |
| Rainfall salinity | 0.322  0.068 | 0.030  0.867 | -0.037  0.836 | 0.156  0.387 | -0.146  0.417 | -0.075  0.680 | 0.201  0.262 | 0.055  0.760 | -0.085  0.640 | -0.066  0.714 | 0.190  0.289 | -0.140  0.436 | -0.025  0.890 | 0.004  0.984 | -0.120  0.507 |

Table S14 Cyanobacterial BG -11 Medium mineral concentrations.

| Mineral | Content (mol 10^-3^ l) | Content (g 10^-3^ l) | Chemicals |
| --- | --- | --- | --- |
| N | 17.64706 | 247.0588 | NaNO_3_ |
| P | 0.229647 | 7.119072 | K_2_HPO_4_ |
| K | 0.459295 | 17.95843 | K_2_HPO_4_ |
| Ca | 0.244865 | 9.794586 | CaCl_2_·2H_2_O |
| Fe | 0.022901 | 1.279008 | Ferric ammonium citrate |
| Mn | 0.009151 | 0.503286 | MnCl_2_·4H_2_O |
| Zn | 0.000772 | 0.050491 | ZnSO_4_·7H_2_O |
| Cu | 0.000316 | 0.020066 | CuSO_4_·5H_2_O |
| Co | 0.000168 | 0.009918 | Co(NO_3_)_2_·6H_2_O |


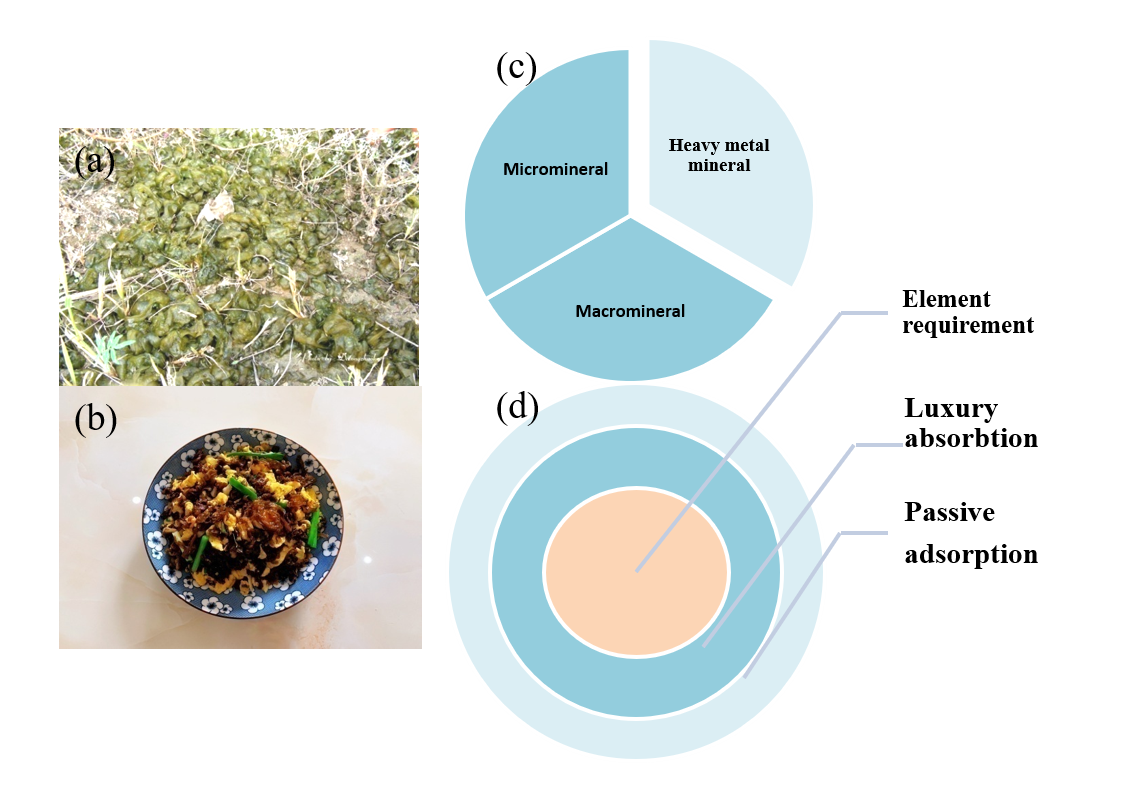


Figure S1 ***N. commune* in China.**

(a) *N. commune* (active state) growing in the natural environment, (b) *N. commune* as traditional food in China, (c, d) mineral elements in *N. commune*.


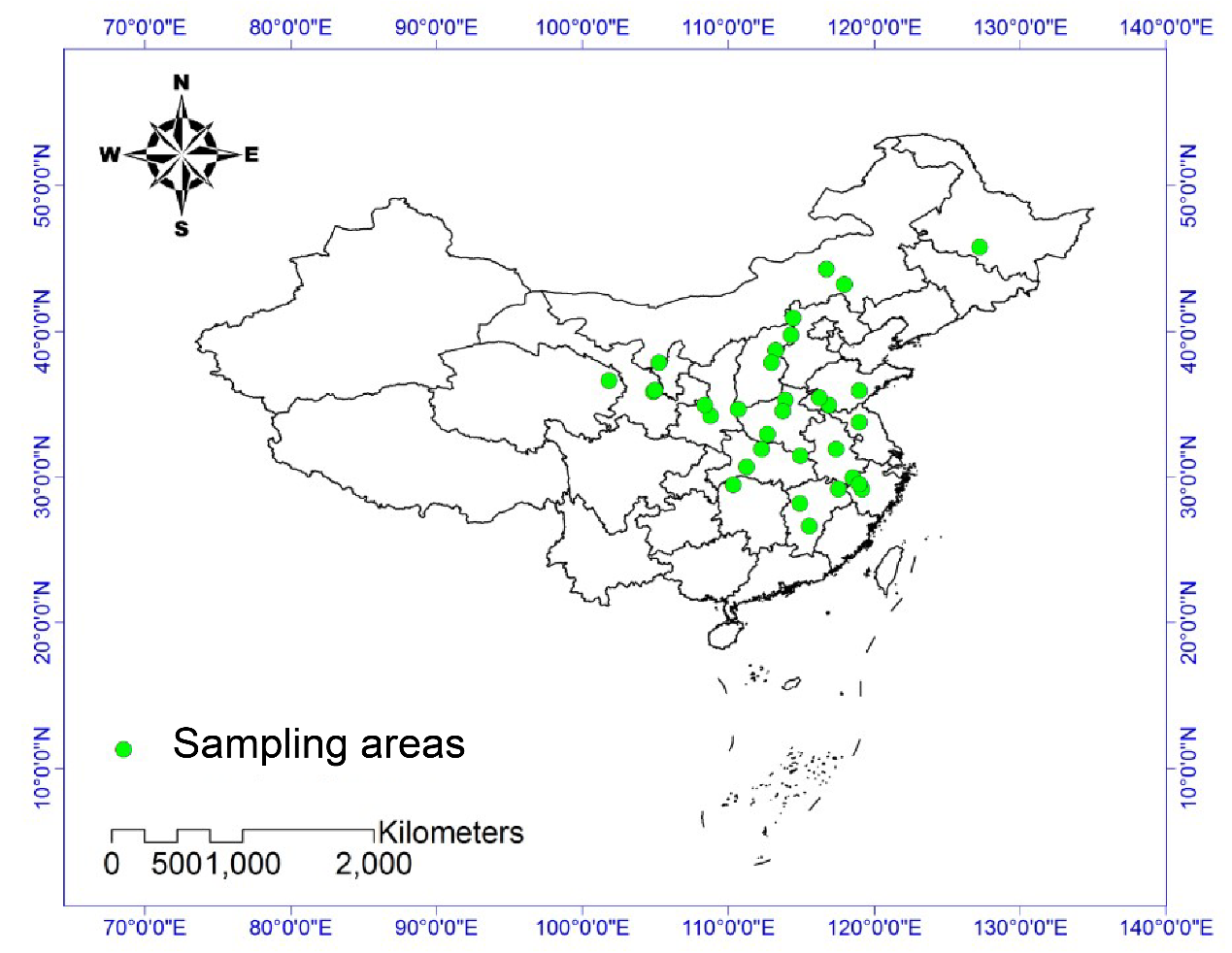


**Figure S2 Map of the China showing all the sampling areas in this study.**





**Figure S3 The average concentration of 15 mineral elements in *N. commune* in China mainland.** Black bars and soil squares are the geometric means on mass and atom basis, respectively, (a) macroelements (N, Ca, K, Fe, P), (b) microelements (Mn, Zn, Cu, Co, Se), (c) heavy metals (Pb, Cr, As, Cd, Hg).


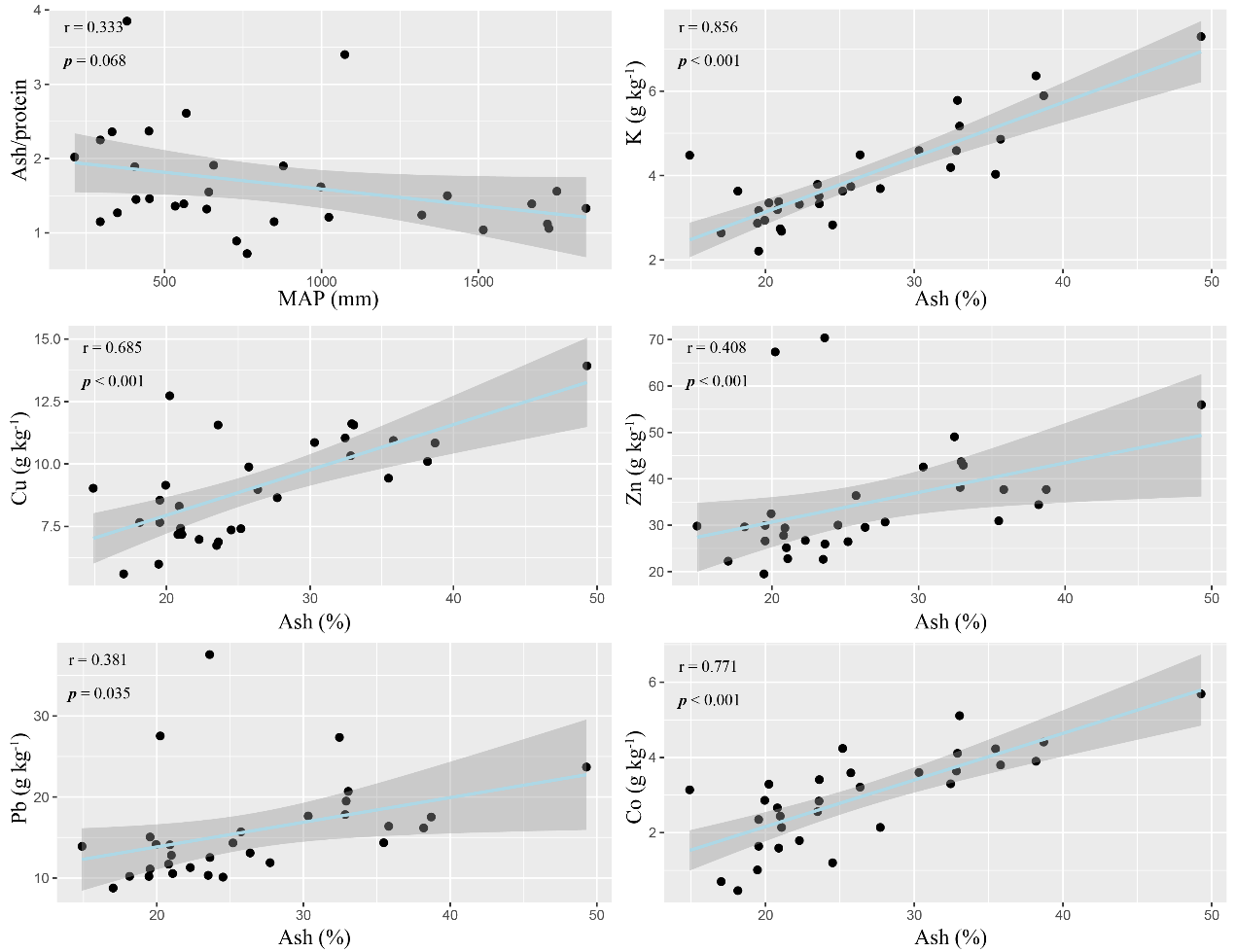


Figure S4 Trends of Ash/Protein in *N. commune* along the MAP gradients in China and relationships between the content of ash and 5 minerals (K, Cu, Zn, Co, Pb).


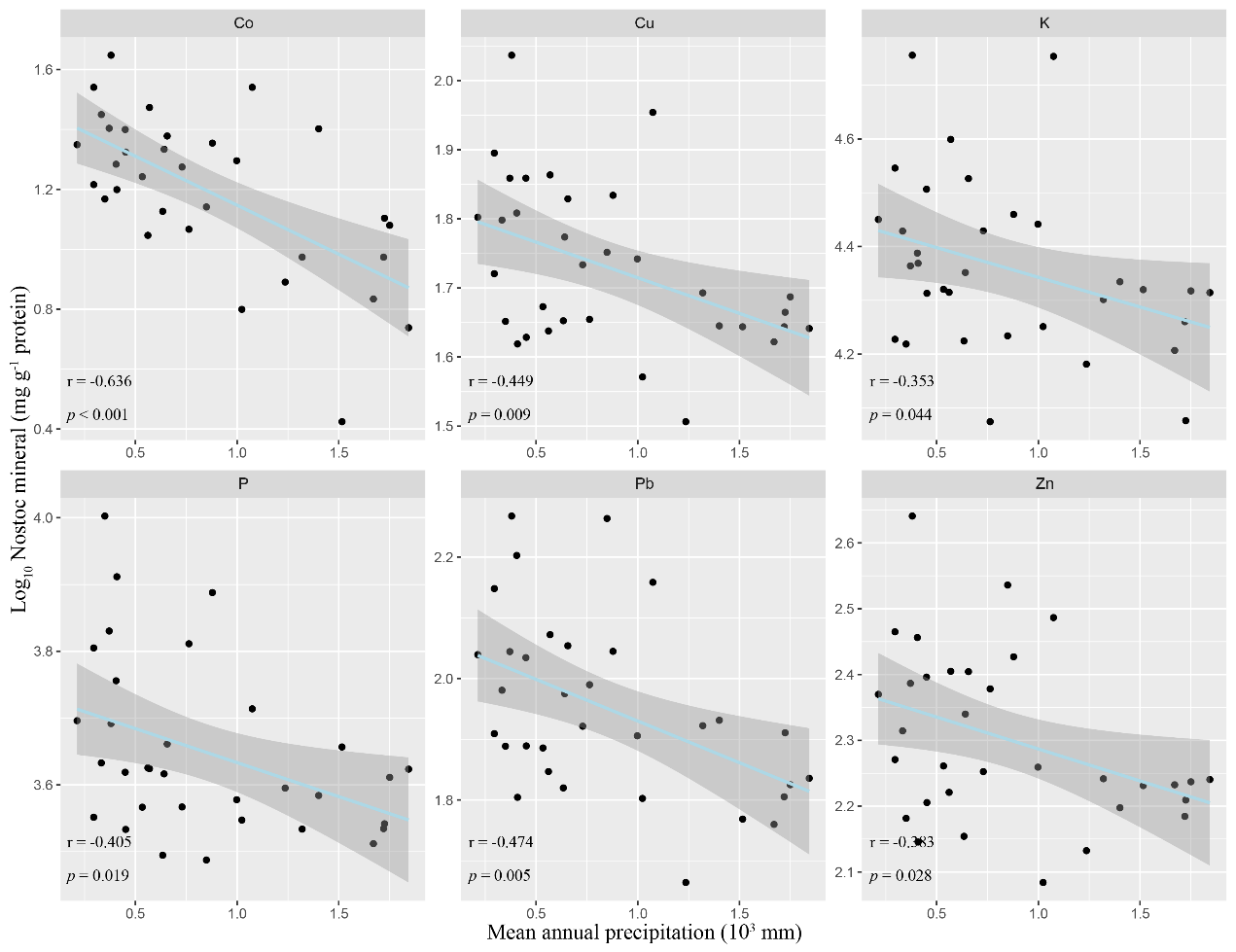


Figure S5 Linear regressions of 6 minerals (K, P, Cu, Zn, Co, Pb) in *N. commune* along the MAP gradients in China (p < 0.05).
